# MiR-212 value in prognosis and diagnosis of cancer and its association with patient characteristics: a systematic review and meta-analysis

**DOI:** 10.1186/s12935-022-02584-0

**Published:** 2022-04-26

**Authors:** Sara Raji, Mehrdad Sahranavard, Mahdi Mottaghi, Amirhossein Sahebkar

**Affiliations:** 1 Persian Cohort Research Center, Mashhad University of Medical Sciences, Mashhad, Iran; 2 School of Pharmacy, Mashhad University of Medical Sciences, Mashhad, Iran; 3 Student Research Committee, School of Pharmacy, Mashhad University of Medical Sciences, Mashhad, Iran; 4 Kidney Transplantation Complications Research Center, Mashhad University of Medical Sciences, Mashhad, Iran; 5 Biotechnology Research Center, Pharmaceutical Technology Institute, Mashhad University of Medical Sciences, Mashhad, Iran; 6 Applied Biomedical Research Center, Mashhad University of Medical Sciences, Mashhad, Iran

**Keywords:** miR-212, miRNA-212, microRNA-212, Prognosis, Diagnosis, Cancer, Neoplasm

## Abstract

**Background:**

Delayed cancer diagnosis and inefficient cancer prognosis determination are problems faced in cancer diagnosis and treatment. MicroRNAs (miRs), especially miR-212, have shown a promise in cancer diagnosis and prognosis. Herein, we performed a systematic review and meta-analysis to assess the prognostic and diagnostic value of miR-212 level in cancer and evaluated its association with patient characteristics.

**Methods:**

A fully electronic literature search using related keywords was performed in PubMed, Scopus, Web of Science, Embase, and ScienceDirect databases by June 6, 2021, with no time or language restriction. Meta-analysis was performed to pool survival prognosis data using hazard ratio (HR), association using odds ratio (OR), and diagnostic data using sensitivity, specificity, and diagnostic odds ratio (DOR). Sub-group analysis and meta-regression were performed as appropriate.

**Results:**

Results of 28 studies on 1880 patients showed a poor cancer prognosis with high levels of miR-212 in pancreatic ductal adenocarcinoma (PDAC, HR = 2.451 [1.447–4.149]), and a poor cancer prognosis with low levels of miR-212 in other cancers (HR = 2.514 [2.162–2.923]). Higher alpha-fetoprotein (AFP) level and Edmondson-Steiner grade were factors associated with miR-212 low level incidence. Diagnostic odds ratio 10.688 (3.644–31.348) and SROC AUC of 0.84 confirmed high diagnostic performance of miR-212.

**Conclusion:**

Our systematic review and meta-analysis results confirm miR-212 high value in cancer prognosis and diagnosis. High level of miR-212 showed poor prognosis in PDAC and low level of miR-212 showed poor prognosis in other cancers. in conclusion, miR-212 could be a novel potential biomarker in cancer diagnosis and prognosis.

**Supplementary Information:**

The online version contains supplementary material available at 10.1186/s12935-022-02584-0.

## Introduction

Cancer is a leading cause of death by causing about 10 million deaths in 2020 worldwide [1]. One potential justification for this high mortality rate is the delayed diagnosis; the highest mortality rates belong to lung, colorectal, hepatic, stomach, and breast cancers, all of which usually present with vague symptoms, especially in the early stages [1, 2]. In addition to delayed diagnosis, ineffective methods to determine the cancer prognosis could cause management flaws leading to under- or over-treatment of patients, both of which impose an economic burden on the patients and health system.

MicroRNAs (miRs) are a main member of small non-coding mRNAs; they act as modulatory tools that work post-transcriptionally to regulate mRNA translation by attaching to the 3'-untranslated region [3, 4]. Genetic codes of miR-212 are located on chromosome 17p13.3. MiR-212 can regulate the cell cycle, proliferation, differentiation, and apoptosis; thus, it can have an oncogenic function or act as a tumor suppressor gene [4]. miRs dysregulation is shown in several cancers, affects patient prognosis and treatment outcomes [2]. miR-212 is one of them which is shown to be altered in hepatocellular carcinoma (HCC), gastric carcinoma, pancreatic ductal adenocarcinoma (PDAC), colorectal carcinoma (CRC), breast cancer, prostate cancer, renal cell carcinoma (RCC), non-small cell lung cancer (NSCLC), etc. Some of these studies assessed prognostic value by finding correlations between miR-212 levels with patient survival, disease-free survival, and recurrence-free survival. Some studies proposed diagnostic power for miR-212 by calculating sensitivity, specificity, and other related factors diagnostic measures. Herein, we systematically reviewed the literature to provide a better view of its prognostic and diagnostic roles, and we powered our findings by multiple meta-analyses on data extracted from these studies.

## Methods

A systematic review and meta-analysis were performed and reported according to The Preferred Reporting Items for Systematic reviews and Meta-Analyses (PRISMA) statement [5].

### Search strategy

Without any time or language restriction, we searched Pubmed, Scopus, Web of Science, Embase, and ScienceDirect on June 6, 2021, using Medical Subject Headings (MeSH), entry terms, and related keywords to miR-212 and cancer. The search was ameliorated by hand search in google scholar and references of included studies. The detailed search strategy is provided in Additional file 1.

### Inclusion criteria

Original human research papers were included in this study. For prognostic evaluation papers providing hazard ratio (HR) or enough data to estimate HR of cancer death comparing patients with low and high levels of miR-212 were included, for patient characteristics association with miR-212 level evaluation papers providing odds ratio (OR) or enough data to calculate OR of patient characteristics comparing patients with low and high level of miR-212 were included, and for diagnostic evaluation papers providing numbers of true and false positive and negative samples using miR-212 level as determinant or enough data to estimate these were included in this study.

### Data extraction

First author name, publication year, country of study, type of assessed miR-212, cancer type, tumor stage, Specimen, and miR-212 assay method were extracted from all studies. For articles included in the prognostic evaluation, crude and adjusted HRs were extracted, and in case of no reported HR, it was extracted from the Kaplan–Meier curve using the Guyot method [6]. For association evaluation, the number of cancer patients in each cell of 2 × 2 table dividing patients in two categories of each characteristic in low and high miR-212 level was extracted from studies. Finally, for diagnostic evaluation, numbers of true positive, false positive, false negative, and true negative were extracted from the articles in three types: reported by the study, obtained from the receiver operating characteristic (ROC) curve using Youden index method [7] and obtained from the ROC curve using Index of Union method [8].

### Quality assessment

Quality assessment was performed using the Newcastle–Ottawa scale (NOS) for the assessment of the quality of nonrandomized studies in meta-analyses [9, 10] for studies included for prognostic and association evaluation and QUADAS-2 tool for the quality assessment of diagnostic accuracy studies [11] for studies included for diagnostic evaluation. For NOS, a score < 4 was considered low quality, a score 4–6 was deemed to be medium quality, and a score of > 6 was regarded as high quality. Search (S. R. and M. S.), screening and full-text assessment (S. R. and M. S.), data extraction (S. R. and M. S.), and Quality assessment (S. R. and M. M.) were all performed by two investigators separately. Disagreements were resolved through discussion or consultation from the third reviewer (A. S.).

### Statistical analysis

Cochran’s Q test and Higgins’s I^2^ and p-value [12, 13] were used to evaluate heterogenicity. In case of low heterogenicity defined by I^2^ < 50 and p-value > 0.05 fixed-effects model was used to pool data; in other conditions, random-effects model was applied. In order to solve observed inconsistency in the included studies email was sent to the corresponding author.

For prognostic evaluation, Comprehensive Meta Analysis software (Version 3.3.070, November 20, 2014) was used to calculate pooled HR. For studies not reporting HR, we digitized the Kaplan–Meier curve using Web Plot Digitizer [14] and estimated HR using coxph function of survival package (Version 2.42-3) using R (Version 4.1.1) in RStudio (Version 1.4.1717) on estimated individual patient data provided by R code in R studio based on the algorithm published by Guyot et al. [6]. For overall survival sub-group analysis and meta-regression were used to find the source of heterogenicity. Sensitivity analysis was performed through one-study-removed analysis, which calculates the pooled effect size of studies after omitting one study each time. Publication bias was assessed using Begg’s funnel plot [15] and Begg’s [15] and Eager’s [16] tests. Nonsignificant p-value of Begg’s and Eager’s tests shows no publication bias. Also, due to the observed different effect of pancreatic cancer on miR-212, publication bias assessment was also performed on studies after removing studies on pancreatic cancer.

For association evaluation of patient characteristics and low miR-212 level incidence, OR calculation and pooling from the number of patients based on characteristics in low miR-212 and high miR-212 was performed using Comprehensive Meta-Analysis software.

For diagnostic evaluation, the numbers of true positive, false positive, false negative and true negative were extracted from articles or calculated using article-provided sensitivity and specificity. To lower the heterogenicity and threshold effect induced by various optimal cut-off determination methods used in different studies, sensitivity and specificity and number of true and false positive and negative were also extracted based on the numbers obtained by Web Plot Digitizer from ROC curves utilizing two methods: higher Youden Index [7], which is the most commonly used method to determine optimal cut-off leading to the point that has the highest summation of sensitivity and specificity, and lower Index of Union [8] which selects the point where sensitivity and specificity are both most near to area under the curve (AUC) and thus to each other [8]. Meta-analysis of Diagnostic and Screening Tests (Meta-DiSc^®^) software (Version 1.4, Madrid, Spain) was used to pool the studies [17]. Pooled sensitivity, specificity, positive likelihood ratio (PLR), negative likelihood ratio (NLR), and diagnostic odds ratio (DOR) and 95% confidence intervals were calculated using the DerSimonian-Laird method. ROC plane and summary ROC (SROC) curve were drawn. The Moses-Shapiro-Littenberg model was used to investigate the constancy of DOR. Not significant p-value of the Moses-Shapiro-Littenberg model shows a constant DOR; thus, symmetrical curve in SROC curve is the preferred curve; otherwise, asymmetrical curve is preferred. SROC AUC curve was drawn applying the exponential of the constant of the Moses-Shapiro-Littenberg model. To investigate the threshold effect, which mainly happens on account of applying different cut-offs in different studies, “Spearman correlation coefficient between the logit of sensitivity and logit of 1-specificity” was calculated [17]. No significant and considerable positive Spearman correlation shows no threshold effect. For further assessment of miR-212 diagnostic value in cancer, Fagan’s nomogram was applied using function nomogram in UncertainInterval package (Version 0.7.0), considering pretest probability of 0.2. Results of Fagan’s nomogram show probability of disease for an individual with a positive test in a population with a prevalence of 20% and probability of disease for an individual with negative test in the same population. To assess publication bias among studies included in the diagnostic evaluation, Deek's funnel plot was drawn and assessed using metabin, metabias, and funnel functions of meta package (Version 4.19-1) in RStudio.

## Results

Performed search provided 874 records, including 173 articles from Pubmed, 295 from Scopus, 187 from Web of Science, 192 from Embase, and 27 from Science Direct. After the removal of duplicate records, 521 articles were left. Title and abstract of articles were screened, resulting in the exclusion of 470 studies. The full textes of 51 remaining articles were assessed resulting in exclusion of 21 articles (12 articles due to not reporting OR and HR or clinical signifance of miR-212, 8 articles investigated a set of microRNAs but not miR-212 solely, and one study investigated diagnosis of the presence of metastasis instead of cancer). Ultimately 28 studies were included in the systematic review and meta-analysis.

Totally 1880 patients from 28 studies were included in this systematic review and meta-analysis. Pooled analysis of prognostic dimension of miR-212 on cancer survival was performed on 1479 patients from 18 studies, pooled analysis of the association between low miR-212 level incidence and patient characteristics were performed on 1438 patients from 19 studies, and pooled analysis of the diagnostic value of miR-212 in cancer diagnosis was performed on 416 patients from 8 studies.

**Fig. 1:**
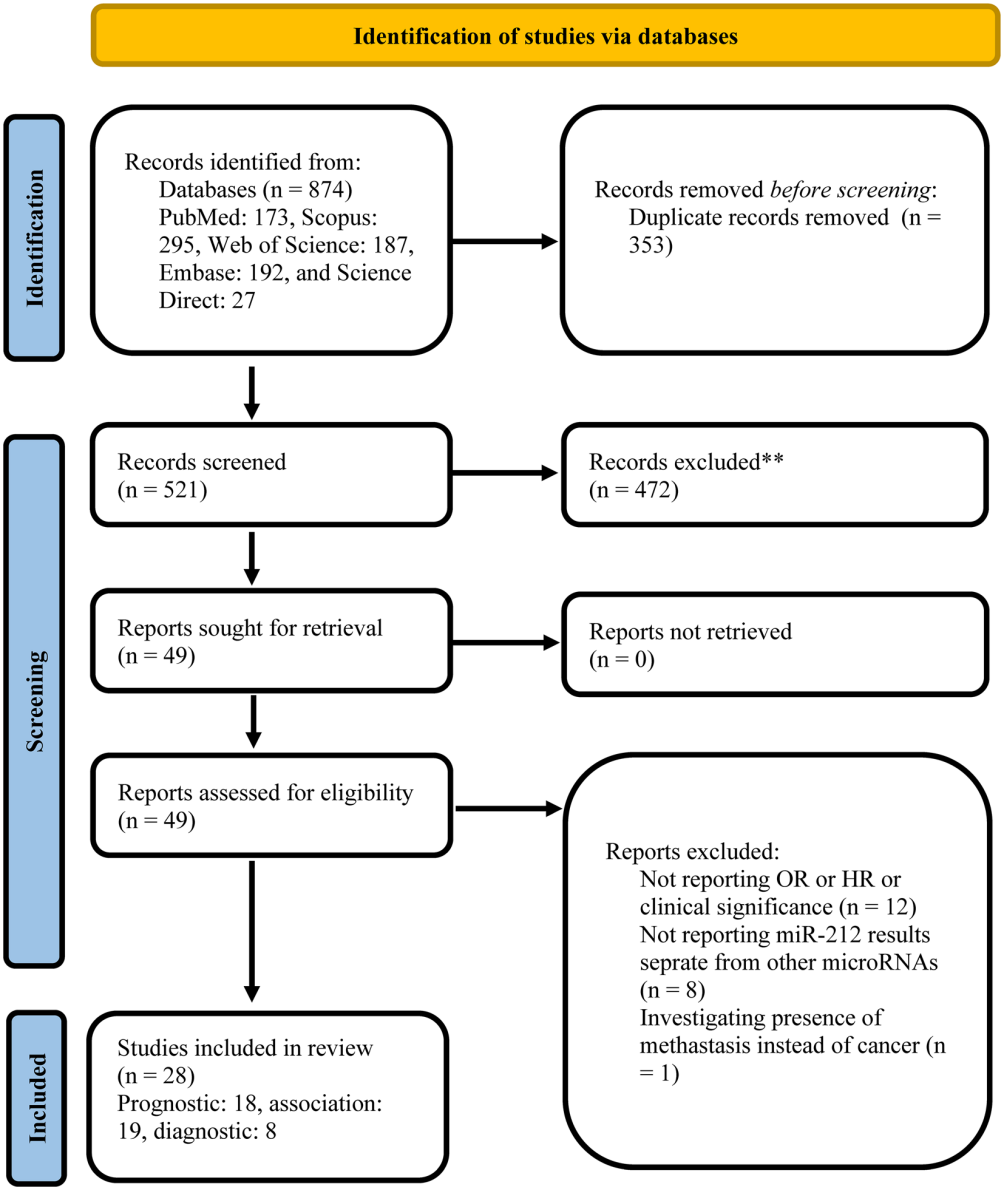
PRISMA flow diagram of the selection process [59]

Studies included in the prognostic evaluation Fig. 1 were performed in China between 2013 and 2020. Five studies were performed on HCC, three on CRC, two on PDAC, two on gastric cancer, and six on other cancers. Studies included in the diagnostic evaluation were performed between 2013 and 2020. Three studies were performed in China, two in Iran, two in the USA, and one in the UK. Each study was performed on a particular organ tumor, and the biofluid samples of plasma, serum, tissue, urine, and bile were used to examine miR-212 levels.

**Fig. 2:**
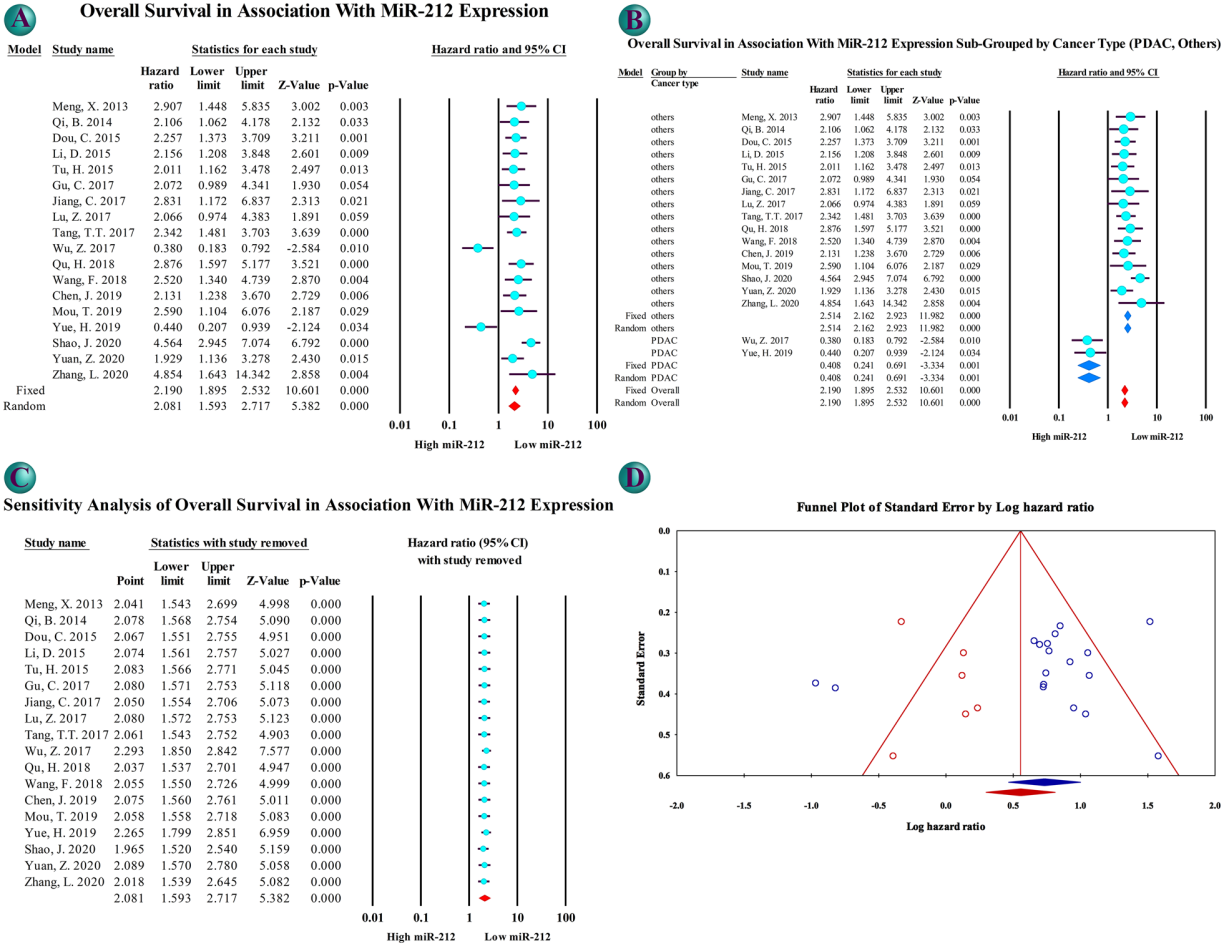
Prognostic evaluation of cancer patients in low miR-212 level patients compared with high miR-212 patients: forrest plot of overall survival (OS) (**A**), forrest plot of OS sub-grouped by cancer type in two groups of Pancreatic ductal adenocarcinoma (PDAC) and other cancer types (**B**), sensitivity analysis of overall survival using one-study-removed method (**C**), and Begg’s funnel plot of publication bias (**D**)

The prognostic dimension of miR-212 in cancer patients was investigated using HR, showing higher mortality of low miR-212 patients compared to high miR-212 patients (Table 2A). HR for overall survival (OS) was 2.081 (1.593, 2.717) based on the data of 1479 patients from 18 studies showing higher mortality in low miR-212 patients (Fig. 2A). The source of heterogenicity observed by I^2^ = 68.884 was investigated through sub-group analysis (Table 2A and Additional file 2: Figures S5–S13). In the analysis sub-grouped by cancer type, I^2^ was 0 for HCC, CRC, and PDAC. In all studies, lower miR-212 showed poorer prognosis, but in two studies on PDAC; so, another sub-group analysis was performed dividing studies in PDAC and other cancers where I^2^ was 0 for both sub-groups and pooled HR was 0.408 (0.241, 0.691) for PDAC and 2.514 (2.162, 2.923) for other cancers (Fig. 2B). In meta-regression analysis, significant regression was observed when studies were tagged by cancer type and sample size. Larger sample size was associated with higher mortality. Also, pooled HR for adjusted overall survival, disease-free survival (DFS), adjusted disease-free survival, and recurrence-free survival (RFS) were all higher than 1.8 and significant (Table 2A and Additional file 2: Figures S14–S17). Sensitivity analysis did not show a considerable difference in pooled effect size after removing any study; however, omitting studies on PDAC resulted in a slight increase in HR (Fig. 2C). NOS results showed high quality of all included studies in prognostic and association evaluation (Table 1). To investigate publication bias, Begg's funnel plot (Fig. 2D) was drawn, and Begg’s and Eager’s tests were performed. Publication bias was rejected by no significant p-value at Begg and Mazumdar rank correlation (p-value = 0.850) and Eager’s regression intercept (p-value = 0.261). To have a broader investigation of publication bias Begg’s and Eager’s tests were performed on studies after removing studies on PDAC, resulting in p-values of 0.242 and 0.909 for Begg’s and Eager’s tests respectively (Additional file 2: Figure S4). Also, Duval and Tweedie’s trim and fill was performed on studies after removing studies on PDAC, confirming study results in cancers other than PDAC by an effect size of 2.514 (2.162–2.923) (Additional file 2: Figure S4).

**Table 1:** Summary of the included studies

A.Studies included in prognostic evaluation and/or association evaluation													
Study ID	MiR-212 type	Country	Sample size	Cancer type	Stage	Specimen	Control	Follow-up (months)	Assay	Cut-off point	Experiment type	NOS score	Survival results
Meng, X. 2013 [31]	miR-212	China	180	CRC	I-III	Tissue	Normal tissue	96	RT- qPCR	Median	In vitro, in vivo	10	OS, DFS
Qi, B. 2014 [32]	miR-212	China	46	EC	0-III	Tissue	Normal tissue	96	RT-qPCR	Median	–	8	OS
Dou, C. 2015 [33]	miR-212	China	95	HCC	I-IV	Tissue	Normal tissue	60	RT-qPCR	Mean	In vitro, in vivo	10	OS, DFS
Li, D. 2015 [34]	miR-212	China	71	GC	0-IV	Tissue	Normal tissue	120	RT-qPCR	Median	In vitro, in vivo	8	OS
Tu, H. 2015 [35]	miR-212	China	86	HCC	I-IV	Tissue	Normal tissue	60	RT-qPCR	Median	In vitro	10	OS, DFS
Gu, C. 2017 [36]	miR-212	China	60	RCC	I-IV	Tissue	Normal tissue	60	RT-qPCR	Mean	In vitro, in vivo	9	OS, RFS
Jiang, C. 2017 [37]	miR-212	China	73	NPC	I-IV	Tissue	Normal tissue	60	RT-qPCR	Median	–	10	OS, DFS
Lu, Z. 2017 [38]	miR-212-5p	China	125	TNBC	NR	Tissue	Normal tissue	136	RT-qPCR	NR	In vitro, in vivo	10	OS, DFS
Tang, T. 2017 [39]	miR-212	China	115	NSCLC	I-IV	Tissue	Normal tissue	60	RT-qPCR	Median	In vitro	9	OS
Wu, Z. 2017 [40]	miR-212	China	45	PDAC	I-IV	Tissue	Normal tissue	36	FISH	Median	–	8	OS
Zhou, Y. 2017 [41]	miR-212	China	58	PCa	NR	Tissue	Normal tissue	NA	RT-qPCR	NR	In vitro, in vivo	9	NA
Qu, H. 2018 [42]	miR-212	China	72	PCa	NR	Tissue	Other individuals	156	RT-qPCR	Median	In vitro	9	OS
Tong, Z. 2018 [43]	miR-212	China	48	RCC	I-IV	Tissue	Normal tissue	NA	RT-qPCR	Mean	In vitro	9	NA
Wang, F. 2018 [44]	miR-212	China	80	HCC	I-IV	Serum	Other individuals	36	RT-qPCR	Median	–	9	OS
Chen, J. 2019 [45]	miR-212-3p	China	83	HCC	I-III	Tissue	Normal tissue	66	RT-qPCR	Median	–	10	OS, RFS
Mou, T. 2019 [46]	miR-212	China	53	CRC	I-IV	Tissue	Normal tissue	60	RT-qPCR	Median	In vitro	10	OS
Yue, H. 2019 [47]	miR-212	China	41	PDAC	I-IV	Tissue	Normal tissue	25	RT-qPCR	Middle of the range	In vitro	10	OS
Azar, M. 2020 [48]	miR-212	Iran	30	TSCC	I-IV	Tissue	Normal tissue	NA	RT-qPCR	NR	–	10	NA
Kang, Y. 2020 [49]	miR-212	China	30	NPC	I-IV	Tissue	Normal tissue	NA	RT-qPCR	NR	In vitro	10	NA
Shao, J. 2020 [50]	miR-212	China	110	GC	I-IV	Serum	Other individuals	53	RT-qPCR	Median	In vitro	10	OS
Yuan, Z. 2020 [51]	miR-212-3p	China	90	HCC	I-IV	Tissue	Normal tissue	60	RT-qPCR	NR	In vitro	9	OS
Zhang, L. 2020 [52]	miR-212-3p	China	63	HGSOC	I-IV	Tissue	NR	130	RT-qPCR	Median	-	9	OS, DFS

*B * Studies included in diagnostic evaluation										
Study ID	miR-212 type	Country	Cancer type	Stage	Normalizer	Control source	Assay	Sample type	AUC (95% CI)	Cancer
Miah, S. 2012 [53]	miR-212	UK	BC (UCC)	pTa- pT4	NR	Healthy individuals	qRT-PCR	Urine	NR	Higher miR
Cote, G. 2014 [54]	miR-212	USA	PDAC	I-IV	NR	CP or BBD	qRT-PCR	Plasma	0.9	Higher miR
								Bile	0.981	Higher miR
Ramalinga, M. 2015 [55]	miR-212	USA	Pca	NR	NR	Healthy individuals	qRT-PCR	Serum	0.66 (0.53–0.78)	Lower miR
Bagheri, A. 2016 [56]	miR-212	Iran	NSCLC	I-IV	ncRNAs	Healthy individuals	qRT-PCR	Sputum	0.69 (0.53–0.85)	Lower miR
								Tissue	0.62 (0.47–0.77)	NR
Damavandi, Z. 2016 [57]	miR-212	Iran	BrC	I-III	5srRNA	Normal tissue	qRT-PCR	Tissue	0.63	Lower miR
Wang, F. 2018 [44]	miR-212	China	HCC	I-IV	cel-miR-39	Healthy individuals	qRT-PCR	Serum	0.706 (0.63–0.73)	Lowe r miR
Pu, X. 2020 [58]	exmiR-212-3p	China	PC	I-IV	NR	Healthy individuals	TCLN biochip	Peripheral blood plasma	0.599	NR
Shao, J. 2020 (50)	miR-212	China	GC	I-IV	NR	Healthy individuals	qRT-PCR	Serum	0.96	Lower miR

*NR* not reported, *NA* not applicable, *CRC* colorectal cancer, *EC* esophageal cancer, *HCC* hepatocellular carcinoma, *GC* gastric cancer, *RCC* renal cell carcinoma, *NPC* nasopharyngeal carcinoma, *TNBC* triple-negative breast cancer, *NSCLC* non-small cell lung cancer, PDAC pancreatic ductal adenocarcinoma, *PCa* prostate cancer, *TSCC* tongue squamous cell carcinoma, *HGSOC* high-grade serous ovarian cancer, *OS* overall survival, *DFS* disease-free survival, *RFS* recurrence-free survival, *qRT-PCR* quantitative real-time PCR, *R* reported by the article, *C* curve, *BC* bladder cancer, *UCC* urothelial cell carcinoma, *BrC* breast cancer, *PC* pancreatic cancer, *CP* chronic pancreatitis, *BBD* benign biliary disorders, *TCLN* tethered cationic lipoplex nanoparticle biochip

Pooled association between low miR-212 level incidence and patient characteristics is shown in Table 2B and Additional file 2: Figures S18–S37. Higher tumor (T), nodes (N), and metastases (M) (TNM) stage was associated with the incidence of low miR-212 level; however, larger tumor size and distant metastasis did not show any association with miR-212 level. Higher serum alpha-fetoprotein (AFP) level showed a significant association with lower miR-212 level. The association was significant in pooling all studies or pooling studies considered 400 mg serum AFP level as cut-off. While pooling studies considering 200 as serum AFP level cut-off did not show a significant association. Presence of venous infiltration and higher Edmondson-Steiner grade were other factors being associated with low miR-212 level incidence.

**Table 2:** Prognostic, association, and diagnostic evaluation of miR-212

A. Prognostic evaluation							
Sub-group	N of studies	N of patients	Pooled hazard ratio (HR) (95% CI)		*p*-value	Heterogeneity	
			Fixed	Random		I^2^	*p*-value
Overall survival							
Overall	18	1479	2.190 (1.895–2.532)	2.081 (1.593–2.717)	< 0.001	68.9	< 0.001
Publication year					0.226	77.07	< 0.001
< 2017	5	478	2.234 (1.719–2.904)	2.234 (1.719–2.904)	< 0.001	0.00	0.947
2017	5	418	1.715 (1.277–2.303)	1.611 (0.811–3.200)	0.174	79.7	0.001
> 2017	8	583	2.463 (1.986–3.055)	2.284 (1.433–3.641)	0.001	77	< 0.001
Sample size					0.021	71.15	< 0.001
< 75	9	515	1.703 (1.336–2.171)	1.722 (1.002–2.961)	0.049	79.2	< 0.001
> 75	9	964	2.518 (2.102–3.016)	2.498 (2.042–3.055)	< 0.001	18.1	0.282
Cancer type					< 0.001	0.00	0.756
HCC	5	434	2.145 (1.681–2.736)	2.145 (1.681–2.736)	< 0.001	0.00	0.974
CRC	3	293	2.508 (1.622–3.878)	2.508 (1.622–3.878)	< 0.001	0.00	0.805
PDAC	2	86	0.408 (0.241–0.691)	0.408 (0.241–0.691)	0.001	0.00	0.785
Gastric	2	181	3.474 (2.450–4.927)	3.216 (1.545–6.696)	0.002	75.6	0.043
Cancer type					< 0.001	0.28	0.438
PDAC	2	86	0.408 (0.241–0.691)	0.408 (0.241–0.691)	0.001	0.00	0.785
Others	16	1393	2.514 (2.162–2.923)	2.514 (2.162–2.923)	< 0.001	0.00	0.660
Stage					0.484	80.14	< 0.001
I-IV	12	902	2.121 (1.779–2.528)	1.944 (1.304–2.898)	0.001	79.3	< 0.001
Others	6	577	2.346 (1.816–3.031)	2.346 (1.816–3.031)	< 0.001	0.00	0.942
Follow-up period					0.484	80.14	< 0.001
< = 60	11	848	2.074 (1.735–2.478)	1.833 (1.214–2.767)	0.004	80.3	< 0.001
> 60	7	631	2.438 (1.900–3.129)	2.438 (1.900–3.129)	< 0.001	0.00	0.825
Cut-off point					0.389	78.91	< 0.001
Median	13	1068	2.412 (2.035–2.859)	2.320 (1.713–3.141)	< 0.001	66.5	< 0.001
Others	5	411	1.693 (1.282–2.235)	1.568 (0.915–2.690)	0.102	71.9	0.007
Specimen					0.097	73.08	< 0.001
Tissue	16	1289	1.973 (1.684–2.311)	1.933 (1.473–2.537)	< 0.001	64.3	< 0.001
Serum	2	190	3.763 (2.626–5.393)	3.549 (1.996–6.308)	< 0.001	56.4	0.130
MiR-212 type					0.836	80.34	< 0.001
miR-212	14	1127	2.189 (1.861–2.574)	2.001 (1.435–2.791)	< 0.001	75.2	< 0.001
miR-212-3p	3	227	2.228 (1.558–3.188)	2.259 (1.525–3.344)	< 0.001	12.8	0.318
miR-212-5p	1	125	2.066 (0.974–4.383)		–		–
Adjusted overall survival							
Overall	5	540	2.121 (1.561–2.881)	2.121 (1.561–2.881)	< 0.001	0.00	0.618
Disease-free survival							
Overall	5	559	2.812 (2.138–3.700)	2.812 (2.138–3.700)	< 0.001	0.00	0.892
Adjusted disease-free survival							
Overall	2	181	2.059 (1.360–3.115)	2.278 (1.085–4.786)	0.030	63.1	0.100
Recurrence-free survival							
Overall	2	143	1.842 (1.240–2.737)	1.842 (1.240–2.737)	0.002	0.00	0.742

B. Association evaluation							
Sub-group	N of studies	N of patients	Pooled odds ratio (OR) (95% CI)		*p*-value	Heterogeneity	
			Fixed	Random		I^2^	*p*-value
Gender (male/female)	13	1026	1.012 (0.766–1.337)	0.993 (0.729–1.353)	0.931	15	0.293
Age (old/young)	18	1394	0.819 (0.652–1.028)	0.819 (0.652–1.028)	0.085	0.00	0.899
Metastasis (yes/no)	4	228	2.521 (1.417–4.486)	3.110 (0.624–15.508)	0.166	86.2	< 0.001
Lymphatic metastasis (yes/no)	3	195	4.614 (1.958–10.873)	4.529 (0.576–35.588)	0.151	62.3	0.071
Tumor size (large/small)	14	1096	1.344 (1.038–1.741)	1.195 (0.635–2.249)	0.580	82.1	< 0.001
Tumor size ([> 5 or ≥ 5]/[< 5 or ≤ 5])	8	712	1.296 (0.942–1.783)	1.455 (0.630–3.364)	0.380	84.7	< 0.001
T stage (III-IV/I-II)	2	222	0.792 (0.401–1.563)	0.515 (0.082–3.212)	0.477	79.7	0.027
TNM stage (III-IV/I-II)	12	786	2.844 (2.057–3.932)	2.719 (1.674–4.415)	< 0.001	51.3	0.020
Differentiation (others/poor)	4	306	0.894 (0.557–1.435)	0.894 (0.556–1.438)	0.642	0.67	0.389
Hepatitis B virus (present/absent)	5	434	0.916 (0.576–1.456)	0.955 (0.545–1.673)	0.711	28.6	0.231
Serum AFP level (high/low)	5	434	2.319 (1.559–3.451)	2.319 (1.559–3.451)	< 0.001	0.00	0.646
Serum AFP level (> 200/ ≤ 200)	2	163	1.620 (0.862–3.047)	1.620 (0.862–3.047)	0.134	0.00	0.642
Serum AFP level (≥ 400/ < 400)	3	271	2.933 (1.759–4.889)	2.933 (1.759–4.889)	< 0.001	0.00	0.892
Number of tumor nodules (≥ 2/1)	3	264	1.216 (0.718–2.060)	1.275 (0.536–3.031)	0.583	62.4	0.070
Cirrhosis (present/absent)	5	434	1.111 (0.748–1.649)	1.118 (0.728–1.718)	0.602	15.3	0.317
Venous infiltration (present/absent)	3	271	1.859 (1.145–3.019)	1.859 (1.145–3.019)	0.012	0.00	0.735
Edmondson-Steiner grade (III-IV/I-II)	4	354	1.757 (1.129–2.735)	1.757 (1.129–2.735)	0.013	0.00	0.532
Venous invasion (positive/negative)	4	320	2.351 (1.460–3.785)	1.772 (0.629–4.996)	0.279	76.9	0.005
Distant metastasis (M1/M0)	3	283	1.401 (0.723–2.714)	0.726 (0.102–5.183)	0.750	85.7	0.001
Histological grade (poor, moderate-well)	2	225	1.231 (0.667–2.273)	1.080 (0.415–2.808)	0.505	45.3	0.176

C. Diagnostic evaluation							
Youden index							
Sub-group	N	Sen	Spe	PLR	NLR	DOR	RDOR, p-value
Overall							
Overall	8	0.72 (0.67–0.76)	0.79 (0.75–0.83)	3.25 (1.85–5.71)	0.38 (0.23–0.63)	10.69 (3.64–31.35)	
I^2^, p-value		91.5, < 0.001	88.1, < 0.001	84.9, < 0.001	90.1, < 0.001	87.3, < 0.001	
Publication year							3.26 (0.00–1.8E5), 0.754
< 2017	5	0.63 (0.55–0.70)	0.79 (0.72–0.85)	3.01 (1.35–6.69)	0.49 (0.29–0.81)	8.69 (2.28–33.08)	
> 2017	3	0.78 (0.72–0.84)	0.79 (0.74–0.84)	3.64 (1.43–9.22)	0.28 (0.10–0.82)	13.41 (1.89–95.26)	
Ethnicity							0.29 (0.00–3.7E3), 0.707
Caucasian	3	0.72 (0.63–0.79)	0.74 (0.66–0.81)	2.45 (0.91–6.54)	0.28 (0.08–1.07)	12.92 (1.41–118.79)	
Asian	5	0.72 (0.66–0.77)	0.81 (0.76–0.85)	3.88 (1.88–8.01)	0.40 (0.21–0.77)	10.80 (2.78–41.86)	
Sample size							0.41 (0.00–1.6E2), 0756
< 50	5	0.64 (0.56–0.72)	0.84 (0.78–0.89)	3.84 (1.58–9.29)	0.46 (0.28–0.77)	9.85 (3.11–31.19)	
> 50	3	0.76 (0.70–0.81)	0.75 (0.69–0.81)	2.93 (1.14–7.56)	0.30 (0.10–0.95)	10.14 (1.24–83.07)	
Sample type							1.05 (0.00–4.2E2), 0.986 (serum vs. others)
Serum	3	0.83 (0.78–0.88)	0.75 (0.69–0.80)	3.07 (1.24–7.58)	0.23 (0.07–0.79)	13.90 (1.79–108.13)	
Plasma	2	0.72 (0.61–0.82)	0.82 (0.74–0.89)	3.92 (2.09–7.34)	0.28 (0.05–1.63)	14.26 (1.63–124.80)	
Tissue	2	0.40 (0.26–0.55)	0.94 (0.83–0.99)	6.26 (1.98–19.80)	0.65 (0.52–0.83)	9.72 (2.63–35.90)	
Index of union							
Overall							
Overall	8	0.74 (0.70–0.78)	0.73 (0.69–0.78)	2.38 (1.48–3.83)	0.37 (0.22–0.64)	7.49 (2.62–21.44)	
I^2^, p-value		87.1, < 0.001	87.6, < 0.001	85.2, < 0.001	87.2, < 0.001	88.8, < 0.001	
Publication year							6.78 (0.00–6.3E5), 0.631
< 2017	5	0.66 (0.59–0.73)	0.71 (0.64–0.78)	1.84 (1.10–3.08)	0.49 (0.29–0.84)	4.67 (1.52–14.33)	
> 2017	3	0.81 (0.75–0.86)	0.75 (0.69–0.80)	3.13 (1.30–7.58)	0.27 (0.09–0.81)	12.11 (1.65–89.01)	
Ethnicity							0.14 (0.00–2.6E3), 0.570
Caucasian	3	0.68 (0.59–0.76)	0.76 (0.67–0.83)	2.53 (0.88–7.22)	0.36 (0.13–1.02)	10.20 (1.26–82.66)	
Asian	5	0.77 (0.72–0.82)	0.73 (0.67–0.78)	2.45 (1.35–4.44)	0.37 (0.18–0.75)	6.95 (1.78–27.18)	
Sample size							0.56 (0.00–3.4E3), 0.844
< 50	5	0.71 (0.63–0.78)	0.71 (0.64–0.77)	1.94 (1.17–3.22)	0.45 (0.27–0.77)	5.30 (1.75–16.09)	
> 50	3	0.76 (0.70–0.81)	0.75 (0.69–0.81)	2.93 (1.14–7.56)	0.30 (0.10–0.95)	10.14 (1.24–83.07)	
Sample type							0.90 (0.00–5.5E3), 0.970
Serum	3	0.81 (0.75–0.86)	0.76 (0.69–0.81)	3.06 (1.20–7.81)	0.27 (0.08–0.84)	11.94 (1.49–95.54)	
Plasma	2	0.79 (0.68–0.88)	0.73 (0.64–0.81)	3.12 (1.06–9.14)	0.26 (0.06–1.22)	12.28 (1.04–144.29)	
Tissue	2	0.54 (0.39–0.69)	0.71 (0.56–0.83)	2.65 (0.44–16.16)	0.63 (0.45–0.89)	4.06 (0.59–27.69)	

The pooled hazard ratio (OR) of survival analysis showing mortality in low miR-212 patients compared to high miR-212 patients (**A**). The pooled odds ratio (OR) showing the association between patient characteristics with the incidence of low miR-212 level (**B**). Diagnostic variables in cancer diagnosis using miR-212 level (**C**), and meta-regression analysis of diagnostic value based on study preferences

*N* number, *Fixed* fixed-effects model, *Random* random-effects model, *Sen* sensitivity, *Spe* specificity, *PLR* positive likelihood ratio, *NLR* negative likelihood ratio, *DOR* diagnostic odds ratio, *RDOR* meta-regression relative diagnostic odds ratio

**Fig. 3:**
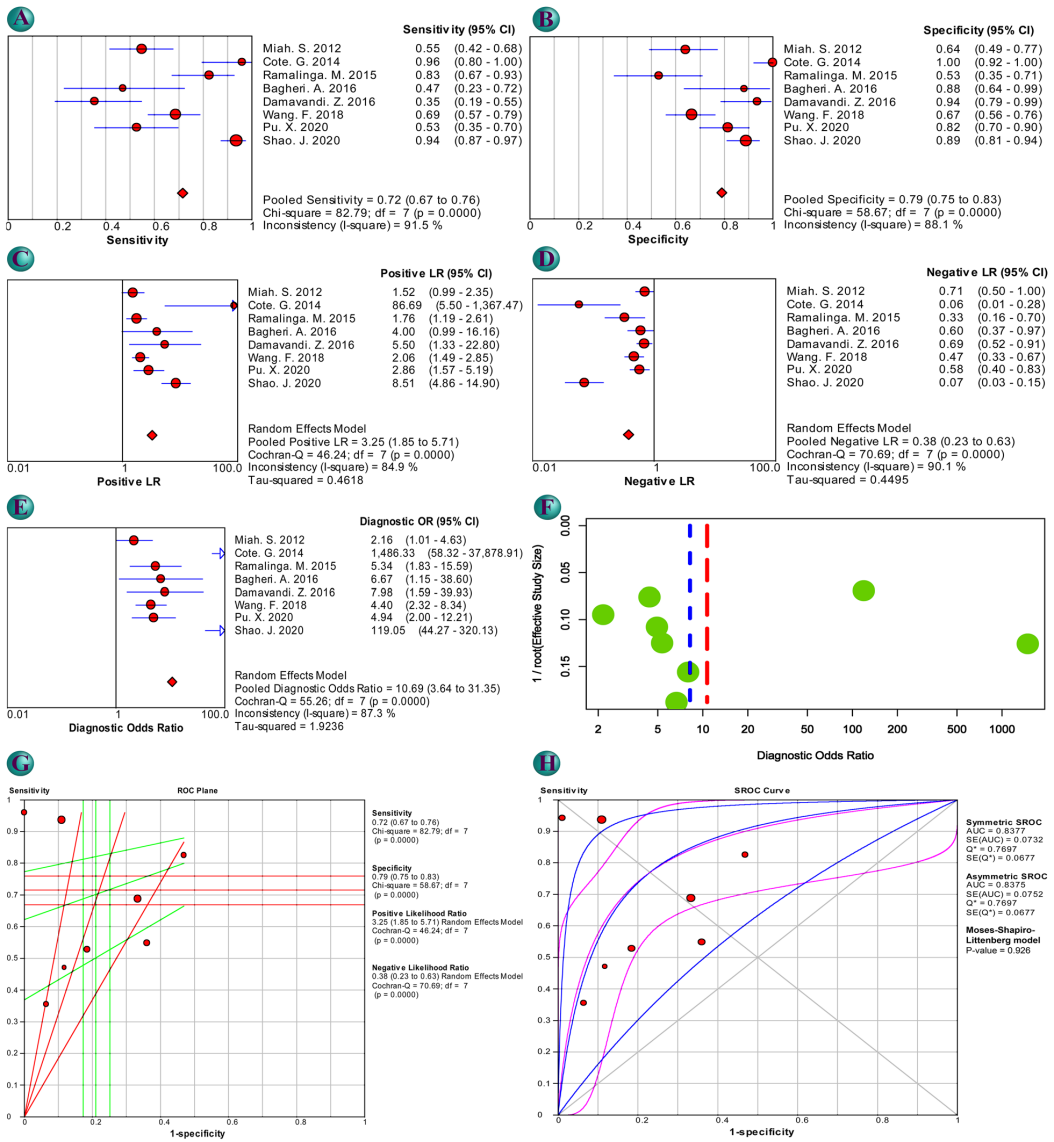
Diagnostic evaluation of cancer using miR-212 level as determinant based on numbers preferably obtained by Youden index: forrest plot of sensitivity (**A**), specificity (**B**), positive likelihood ratio (LR) (**C**), negative LR (**D**), and diagnostic odds ratio (OR) (**E**), Deek's funnel plot (**F**), receiver operating characteristic (ROC) plane (**G**) and Summary ROC (SROC) curve (**H**)

Evaluation of diagnostic value based on the numbers obtained preferably using Youden Index showed pooled values of sensitivity 0.716 (0.669–0.759), specificity 0.790 (0.748–0.827), positive likelihood ratio 3.249 (1.849–5.709), negative likelihood ratio 0.378 (0.226–0.631), and diagnostic odds ratio 10.688 (3.644–31.348) and SROC AUC of 0.84 confirming high diagnostic performance of miR-212 (Table 2C and Fig. 3). Numbers preferably obtained using the Index of Union confirmed the findings (Table 2C and Additional file 2: Figures S39). Results of subgroup analysis and meta-regression are provided in Table 2C. Meta-regression did not show significant regression in any considered factor. Indicated by Spearman correlation coefficient, no positive correlation between the logit of sensitivity and logit of 1-specificity concluded no threshold effect in analysis using numbers preferably obtained by Youden Index (− 0.048, p-value = 0.911) and Index of Union (− 0.762 p-value = 0.028). Results of QUADAS-2 are demonstrated in Additional file 2: Figure S40 and S41. Fagan’s nomogram based on pooled sensitivity and specificity acquired by numbers preferably obtained by Youden Index showed a positive predictive value of 46.2%, meaning 0.46 probability of having cancer in an individual from a tested population with 20% cancer prevalence with a positive miR-212 cancer test (Additional file 2: Figure S42 and S43). Also, the negative post-test result was found to be 8.25% showing an individual from the same population with a negative test result has a probability of 0.08 to be affected by cancer. Deek’s funnel plot did not show any publication bias using numbers obtained preferably by Youden Index (p-value = 0.798, Fig. 3F) and Index of Union (p-value = 0.652, Additional file 2: Figure S39F).

## Discussion

Timely cancer detection and accurate estimation of prognosis are crucial for appropriate patient management. The prognostic and diagnostic value of miRs is trending as they could be our potential solution. miR-212 is one of many miRs with aberrant expression in different cancer patients; Herein, we analyzed its diagnostic accuracy and prognostic value as the existing studies reported valuable but inconsistent results. Given the unique pathophysiology of each cancer and even different subtypes of particular cancer, it would be more helpful to assess miR-212 based on cancer type and subtype; however, due to relatively scarce standard studies on every single cancer, we aimed to evaluate its importance in a metanalytic overview.

Our prognostic assessment showed that the HR of low levels of miR-212 to its high levels was 2.081 (1.593, 2.717, I^2^ = 68.884) in predicting OS. To eliminate heterogeneity, we used subgroup analysis which detected PDAC studies as the heterogeneity source. Subgroup analysis illustrated a poorer prognosis of low miR-212 levels in cancers other than PDAC (HR = 2.514); however, PDAC was an exception (HR = 0.408). But why a specific miR is increased in one cancer and decreased in another? It should be noted that one miR potentially can interact with 200 genes [4]. For instance, the Retinoblastoma (Rb)-1 tumor suppressor gene is the target of miR-212 in PDAC (increased miR-212 suppresses Rb1 that promotes cell growth), but Rb binding protein-2 (RBP2) is the target in gastric carcinoma and HCC (decreased miR-212 upregulates RBP2 which promotes cell growth) [4]. When miR-212 can target tumor suppressor genes or their products, it is theoretically plausible for them to be a cancer biomarker with diagnostic and prognostic value. About PDAC, different studies showed consistent results; Yue et al. observed increased levels of both miR-212 and hypoxia-induced factor-1α (HIF-1α) in PDAC patients [18]. They powered their findings via in vitro analysis of the miR-212 promoter region, which possesses five hypoxia response elements, potentially able to bind HIF-1α. Schultz et al. and Wu et al. also found elevated miR-212 in PDAC patients [4, 19]. Several studies indicated that lower miR-212 is tied to worse outcomes in HCC patients [20–23]. Dou et al. found that HCC-related OS and DFS are predictable via miR-212 levels combined with Forkhead box protein-A1 (FOXA1), which was more reliable than each alone [21]. FOXA1 promotes cell proliferation and apoptosis with an established role in HCC development and post-transcriptionally down-regulated by miR-212-3p [21, 24]. The miR-212 also suppresses the connective tissue growth factor (CTGF) (which promotes tumoral angiogenesis) and histone-H3 lysine-4 demethylase of RBP2 (which is upregulated in HCC) [20, 22, 23]. Similarly, some of the mentioned pathways and some other pathways are declared to be involved in breast cancer, renal cell carcinoma, prostate cancer, nasopharyngeal carcinoma, etc. The relevant targeted genes and their relative function are shown in Additional file 2: Table S1.

Significant pooled association of low miR-212 level incidence with Higher serum AFP level and higher Edmondson-Steiner grade confirm miR-212 value in HCC.

MiRs express abnormally in several cancers; they possess less complex transcriptional and translational modifications than proteins and miRs [2]. They are stable in a wide range of pH and resist degradation with several freezes and thaw procedures [3, 25]. These features make them suitable candidates for cancer diagnosis and treatment. Our diagnostic outcomes resulted from PDAC, HCC, breast cancer, gastric cancer, bladder cancer, prostate cancer, and non-small cell lung cancer studies. In diagnostic evaluation, while using the Youden index, the pooled sensitivity and specificity were 0.716 (0.669–0.759) and 0.790 (0.748–0.827), respectively. DOR is a single number that indicates the diagnostic accuracy of the intended test by dividing PLR to NLR, thus combining both sensitivity and specificity into a single number. Pooled DOR using the Youden index was 10.69 (3.64–31.35), showing that miR-212 could be a potential biomarker in cancer diagnosis. Notably, using multiple miRs as a diagnostic panel could increase diagnostic accuracy to the point that it is applicable in clinical settings, as seen in several studies [26, 27]. Of note, Bagheri et al. reported no additional benefits of using a panel of miRs (miR-223, miR-212, and SNORD37) to detect non-small cell lung cancer, emphasizing the usefulness of miRs as a compound diagnostic tool can be changed based on the type of cancer [3]. This study also encourages future experiments to assess several miRs to find an optimal panel of miRs for each cancer.

Additionally, using miRs based on clinical data (family history, social history, medication history, etc.) and assessing them in specific biofluids (sputum, saliva, bile, urine, etc.) can remarkably increase clinical diagnostic accuracy [25, 27]. Cote et al. showed that miR-212 levels in bile had higher sensitivity, specificity, and DOR than plasma levels for PDAC [27]. Another solution to clinically optimize the diagnostic accuracy of miR-212 is to use it in a high-risk population for each specific cancer [27]. Recently, the diagnostic value of other miRs like miR-375, miR-21, miR-34a, and miR-155 have been studied with relatively similar diagnostic values [28–30]. Future diagnosis of cancer can be revolutionized by proper use of miRs, which accordingly could guide us toward more efficient treatment.

The present review has some limitations: all prognostic articles were from China; thus, more studies are needed in other races to generalize the reported findings. Also, miR-212 diagnostic accuracy and prognostic value differ by type of cancer and even cancer subtypes.

In conclusion, miR-212 can help us diagnose cancers with a prolonged latency period and determine cancer prognosis.

## Supplementary Information

**Additional file 1:** Detailed search strategy.

**Additional file 2:** **Table S1, S2** and **Figure S1–S43**.

## Data Availability

Not applicable.
