# Supplementary material for: MiR-212 value in prognosis and diagnosis of cancer and its association with patient characteristics: a systematic review and meta-analysis
Source: Cancer Cell Int. 2022 Apr 26;22:163. doi: 10.1186/s12935-022-02584-0 (PMC9044851; doi:10.1186/s12935-022-02584-0)
Supplement: Supplementary file 1 — Additional file 1: Detailed search strategy. [file 12935_2022_2584_MOESM1_ESM.docx]

**Search Strategy**

- **Pubmed**
  - **Searched by the authors**

((mir-212) OR (miR-212) OR (miRNA-212) OR (microRNA-212)) AND ((Neoplasms) OR (Neoplasia) OR (Neoplasias) OR (Neoplasm) OR (Tumors) OR (Tumor) OR (Cancer) OR (Cancers) OR (Malignancy) OR (Malignancies) OR (Malignant Neoplasms) OR (Malignant Neoplasm) OR (Neoplasm, Malignant) OR (Neoplasms, Malignant) OR (Benign Neoplasms) OR (Neoplasms, Benign) OR (Benign Neoplasm) OR (Neoplasm, Benign) OR (Carcinoma) OR (Carcinomas) OR (Adenocarcinoma) OR (Adenocarcinomas) OR (Epithelioma) OR (epitheliomas) OR (carcinomatose) OR (carcinomatosis) OR (adenoma) OR (adenomas))

- - **Distinguished by Pubmed**

("mir 212"[All Fields] OR "mir 212"[All Fields] OR "miRNA-212"[All Fields] OR "microRNA-212"[All Fields]) AND ("neoplasm s"[All Fields] OR "neoplasms"[MeSH Terms] OR "neoplasms"[All Fields] OR "neoplasm"[All Fields] OR ("neoplasms"[MeSH Terms] OR "neoplasms"[All Fields] OR "neoplasia"[All Fields] OR "neoplasias"[All Fields]) OR ("neoplasms"[MeSH Terms] OR "neoplasms"[All Fields] OR "neoplasia"[All Fields] OR "neoplasias"[All Fields]) OR ("neoplasm s"[All Fields] OR "neoplasms"[MeSH Terms] OR "neoplasms"[All Fields] OR "neoplasm"[All Fields]) OR ("cysts"[MeSH Terms] OR "cysts"[All Fields] OR "cyst"[All Fields] OR "neurofibroma"[MeSH Terms] OR "neurofibroma"[All Fields] OR "neurofibromas"[All Fields] OR "tumor s"[All Fields] OR "tumoral"[All Fields] OR "tumorous"[All Fields] OR "tumour"[All Fields] OR "neoplasms"[MeSH Terms] OR "neoplasms"[All Fields] OR "tumor"[All Fields] OR "tumour s"[All Fields] OR "tumoural"[All Fields] OR "tumourous"[All Fields] OR "tumours"[All Fields] OR "tumors"[All Fields]) OR ("cysts"[MeSH Terms] OR "cysts"[All Fields] OR "cyst"[All Fields] OR "neurofibroma"[MeSH Terms] OR "neurofibroma"[All Fields] OR "neurofibromas"[All Fields] OR "tumor s"[All Fields] OR "tumoral"[All Fields] OR "tumorous"[All Fields] OR "tumour"[All Fields] OR "neoplasms"[MeSH Terms] OR "neoplasms"[All Fields] OR "tumor"[All Fields] OR "tumour s"[All Fields] OR "tumoural"[All Fields] OR "tumourous"[All Fields] OR "tumours"[All Fields] OR "tumors"[All Fields]) OR ("cancer s"[All Fields] OR "cancerated"[All Fields] OR "canceration"[All Fields] OR "cancerization"[All Fields] OR "cancerized"[All Fields] OR "cancerous"[All Fields] OR "neoplasms"[MeSH Terms] OR "neoplasms"[All Fields] OR "cancer"[All Fields] OR "cancers"[All Fields]) OR ("cancer s"[All Fields] OR "cancerated"[All Fields] OR "canceration"[All Fields] OR "cancerization"[All Fields] OR "cancerized"[All Fields] OR "cancerous"[All Fields] OR "neoplasms"[MeSH Terms] OR "neoplasms"[All Fields] OR "cancer"[All Fields] OR "cancers"[All Fields]) OR ("malign"[All Fields] OR "malignance"[All Fields] OR "malignances"[All Fields] OR "malignant"[All Fields] OR "malignants"[All Fields] OR "malignities"[All Fields] OR "malignity"[All Fields] OR "malignization"[All Fields] OR "malignized"[All Fields] OR "maligns"[All Fields] OR "neoplasms"[MeSH Terms] OR "neoplasms"[All Fields] OR "malignancies"[All Fields] OR "malignancy"[All Fields]) OR ("malign"[All Fields] OR "malignance"[All Fields] OR "malignances"[All Fields] OR "malignant"[All Fields] OR "malignants"[All Fields] OR "malignities"[All Fields] OR "malignity"[All Fields] OR "malignization"[All Fields] OR "malignized"[All Fields] OR "maligns"[All Fields] OR "neoplasms"[MeSH Terms] OR "neoplasms"[All Fields] OR "malignancies"[All Fields] OR "malignancy"[All Fields]) OR ("neoplasms"[MeSH Terms] OR "neoplasms"[All Fields] OR ("malignant"[All Fields] AND "neoplasms"[All Fields]) OR "malignant neoplasms"[All Fields]) OR ("neoplasms"[MeSH Terms] OR "neoplasms"[All Fields] OR ("malignant"[All Fields] AND "neoplasm"[All Fields]) OR "malignant neoplasm"[All Fields]) OR ("neoplasms"[MeSH Terms] OR "neoplasms"[All Fields] OR ("neoplasm"[All Fields] AND "malignant"[All Fields]) OR "neoplasm malignant"[All Fields]) OR ("neoplasms"[MeSH Terms] OR "neoplasms"[All Fields] OR ("neoplasms"[All Fields] AND "malignant"[All Fields]) OR "neoplasms malignant"[All Fields]) OR ("neoplasms"[MeSH Terms] OR "neoplasms"[All Fields] OR ("benign"[All Fields] AND "neoplasms"[All Fields]) OR "benign neoplasms"[All Fields]) OR ("neoplasms"[MeSH Terms] OR "neoplasms"[All Fields] OR ("neoplasms"[All Fields] AND "benign"[All Fields]) OR "neoplasms benign"[All Fields]) OR ("neoplasms"[MeSH Terms] OR "neoplasms"[All Fields] OR ("benign"[All Fields] AND "neoplasm"[All Fields]) OR "benign neoplasm"[All Fields]) OR ("neoplasms"[MeSH Terms] OR "neoplasms"[All Fields] OR ("neoplasm"[All Fields] AND "benign"[All Fields]) OR "neoplasm benign"[All Fields]) OR ("carcinoma"[MeSH Terms] OR "carcinoma"[All Fields] OR "carcinomas"[All Fields] OR "carcinoma s"[All Fields]) OR ("carcinoma"[MeSH Terms] OR "carcinoma"[All Fields] OR "carcinomas"[All Fields] OR "carcinoma s"[All Fields]) OR ("adenocarcinoma"[MeSH Terms] OR "adenocarcinoma"[All Fields] OR "adenocarcinomas"[All Fields] OR "adenocarcinoma s"[All Fields]) OR ("adenocarcinoma"[MeSH Terms] OR "adenocarcinoma"[All Fields] OR "adenocarcinomas"[All Fields] OR "adenocarcinoma s"[All Fields]) OR ("carcinoma"[MeSH Terms] OR "carcinoma"[All Fields] OR "epitheliomas"[All Fields] OR "neoplasms, glandular and epithelial"[MeSH Terms] OR ("neoplasms"[All Fields] AND "glandular"[All Fields] AND "epithelial"[All Fields]) OR "glandular and epithelial neoplasms"[All Fields] OR "epithelioma"[All Fields]) OR ("carcinoma"[MeSH Terms] OR "carcinoma"[All Fields] OR "epitheliomas"[All Fields] OR "neoplasms, glandular and epithelial"[MeSH Terms] OR ("neoplasms"[All Fields] AND "glandular"[All Fields] AND "epithelial"[All Fields]) OR "glandular and epithelial neoplasms"[All Fields] OR "epithelioma"[All Fields]) OR "carcinomatose"[All Fields] OR ("carcinoma"[MeSH Terms] OR "carcinoma"[All Fields] OR "carcinomatosis"[All Fields]) OR ("adenoma"[MeSH Terms] OR "adenoma"[All Fields] OR "adenomas"[All Fields] OR "adenoma s"[All Fields]) OR ("adenoma"[MeSH Terms] OR "adenoma"[All Fields] OR "adenomas"[All Fields] OR "adenoma s"[All Fields]))

**Translations**

**Neoplasms:** "neoplasm's"[All Fields] OR "neoplasms"[MeSH Terms] OR "neoplasms"[All Fields] OR "neoplasm"[All Fields]

**Neoplasia:** "neoplasms"[MeSH Terms] OR "neoplasms"[All Fields] OR "neoplasia"[All Fields] OR "neoplasias"[All Fields]

**Neoplasias:** "neoplasms"[MeSH Terms] OR "neoplasms"[All Fields] OR "neoplasia"[All Fields] OR "neoplasias"[All Fields]

**Neoplasm:** "neoplasm's"[All Fields] OR "neoplasms"[MeSH Terms] OR "neoplasms"[All Fields] OR "neoplasm"[All Fields]

**Tumors:** "cysts"[MeSH Terms] OR "cysts"[All Fields] OR "cyst"[All Fields] OR "neurofibroma"[MeSH Terms] OR "neurofibroma"[All Fields] OR "neurofibromas"[All Fields] OR "tumor's"[All Fields] OR "tumoral"[All Fields] OR "tumorous"[All Fields] OR "tumour"[All Fields] OR "neoplasms"[MeSH Terms] OR "neoplasms"[All Fields] OR "tumor"[All Fields] OR "tumour's"[All Fields] OR "tumoural"[All Fields] OR "tumourous"[All Fields] OR "tumours"[All Fields] OR "tumors"[All Fields]

**Tumor:** "cysts"[MeSH Terms] OR "cysts"[All Fields] OR "cyst"[All Fields] OR "neurofibroma"[MeSH Terms] OR "neurofibroma"[All Fields] OR "neurofibromas"[All Fields] OR "tumor's"[All Fields] OR "tumoral"[All Fields] OR "tumorous"[All Fields] OR "tumour"[All Fields] OR "neoplasms"[MeSH Terms] OR "neoplasms"[All Fields] OR "tumor"[All Fields] OR "tumour's"[All Fields] OR "tumoural"[All Fields] OR "tumourous"[All Fields] OR "tumours"[All Fields] OR "tumors"[All Fields]

**Cancer:** "cancer's"[All Fields] OR "cancerated"[All Fields] OR "canceration"[All Fields] OR "cancerization"[All Fields] OR "cancerized"[All Fields] OR "cancerous"[All Fields] OR "neoplasms"[MeSH Terms] OR "neoplasms"[All Fields] OR "cancer"[All Fields] OR "cancers"[All Fields]

**Cancers:** "cancer's"[All Fields] OR "cancerated"[All Fields] OR "canceration"[All Fields] OR "cancerization"[All Fields] OR "cancerized"[All Fields] OR "cancerous"[All Fields] OR "neoplasms"[MeSH Terms] OR "neoplasms"[All Fields] OR "cancer"[All Fields] OR "cancers"[All Fields]

**Malignancy:** "malign"[All Fields] OR "malignance"[All Fields] OR "malignances"[All Fields] OR "malignant"[All Fields] OR "malignants"[All Fields] OR "malignities"[All Fields] OR "malignity"[All Fields] OR "malignization"[All Fields] OR "malignized"[All Fields] OR "maligns"[All Fields] OR "neoplasms"[MeSH Terms] OR "neoplasms"[All Fields] OR "malignancies"[All Fields] OR "malignancy"[All Fields]

**Malignancies:** "malign"[All Fields] OR "malignance"[All Fields] OR "malignances"[All Fields] OR "malignant"[All Fields] OR "malignants"[All Fields] OR "malignities"[All Fields] OR "malignity"[All Fields] OR "malignization"[All Fields] OR "malignized"[All Fields] OR "maligns"[All Fields] OR "neoplasms"[MeSH Terms] OR "neoplasms"[All Fields] OR "malignancies"[All Fields] OR "malignancy"[All Fields]

**Malignant Neoplasms:** "neoplasms"[MeSH Terms] OR "neoplasms"[All Fields] OR ("malignant"[All Fields] AND "neoplasms"[All Fields]) OR "malignant neoplasms"[All Fields]

**Malignant Neoplasm:** "neoplasms"[MeSH Terms] OR "neoplasms"[All Fields] OR ("malignant"[All Fields] AND "neoplasm"[All Fields]) OR "malignant neoplasm"[All Fields]

**Neoplasm, Malignant:** "neoplasms"[MeSH Terms] OR "neoplasms"[All Fields] OR ("neoplasm"[All Fields] AND "malignant"[All Fields]) OR "neoplasm malignant"[All Fields]

**Neoplasms, Malignant:** "neoplasms"[MeSH Terms] OR "neoplasms"[All Fields] OR ("neoplasms"[All Fields] AND "malignant"[All Fields]) OR "neoplasms, malignant"[All Fields]

**Benign Neoplasms:** "neoplasms"[MeSH Terms] OR "neoplasms"[All Fields] OR ("benign"[All Fields] AND "neoplasms"[All Fields]) OR "benign neoplasms"[All Fields]

**Neoplasms, Benign:** "neoplasms"[MeSH Terms] OR "neoplasms"[All Fields] OR ("neoplasms"[All Fields] AND "benign"[All Fields]) OR "neoplasms, benign"[All Fields]

**Benign Neoplasm:** "neoplasms"[MeSH Terms] OR "neoplasms"[All Fields] OR ("benign"[All Fields] AND "neoplasm"[All Fields]) OR "benign neoplasm"[All Fields]

**Neoplasm, Benign:** "neoplasms"[MeSH Terms] OR "neoplasms"[All Fields] OR ("neoplasm"[All Fields] AND "benign"[All Fields]) OR "neoplasm, benign"[All Fields]

**Carcinoma:** "carcinoma"[MeSH Terms] OR "carcinoma"[All Fields] OR "carcinomas"[All Fields] OR "carcinoma's"[All Fields]

**Carcinomas:** "carcinoma"[MeSH Terms] OR "carcinoma"[All Fields] OR "carcinomas"[All Fields] OR "carcinoma's"[All Fields]

**Adenocarcinoma:** "adenocarcinoma"[MeSH Terms] OR "adenocarcinoma"[All Fields] OR "adenocarcinomas"[All Fields] OR "adenocarcinoma's"[All Fields]

**Adenocarcinomas:** "adenocarcinoma"[MeSH Terms] OR "adenocarcinoma"[All Fields] OR "adenocarcinomas"[All Fields] OR "adenocarcinoma's"[All Fields]

**Epithelioma:** "carcinoma"[MeSH Terms] OR "carcinoma"[All Fields] OR "epitheliomas"[All Fields] OR "neoplasms, glandular and epithelial"[MeSH Terms] OR ("neoplasms"[All Fields] AND "glandular"[All Fields] AND "epithelial"[All Fields]) OR "glandular and epithelial neoplasms"[All Fields] OR "epithelioma"[All Fields]

**epitheliomas:** "carcinoma"[MeSH Terms] OR "carcinoma"[All Fields] OR "epitheliomas"[All Fields] OR "neoplasms, glandular and epithelial"[MeSH Terms] OR ("neoplasms"[All Fields] AND "glandular"[All Fields] AND "epithelial"[All Fields]) OR "glandular and epithelial neoplasms"[All Fields] OR "epithelioma"[All Fields]

**carcinomatosis:** "carcinoma"[MeSH Terms] OR "carcinoma"[All Fields] OR "carcinomatosis"[All Fields]

**adenoma:** "adenoma"[MeSH Terms] OR "adenoma"[All Fields] OR "adenomas"[All Fields] OR "adenoma's"[All Fields]

**adenomas:** "adenoma"[MeSH Terms] OR "adenoma"[All Fields] OR "adenomas"[All Fields] OR "adenoma's"[All Fields]

- **Scopus**
  - **Searched by the authors**

TITLE-ABS-KEY (((mir-212) OR (miR-212) OR (miRNA-212) OR (microRNA-212)) AND ((Neoplasms) OR (Neoplasia) OR (Neoplasias) OR (Neoplasm) OR (Tumors) OR (Tumor) OR (Cancer) OR (Cancers) OR (Malignancy) OR (Malignancies) OR (Malignant Neoplasms) OR (Malignant Neoplasm) OR (Neoplasm, Malignant) OR (Neoplasms, Malignant) OR (Benign Neoplasms) OR (Neoplasms, Benign) OR (Benign Neoplasm) OR (Neoplasm, Benign) OR (Carcinoma) OR (Carcinomas) OR (Adenocarcinoma) OR (Adenocarcinomas) OR (Epithelioma) OR (epitheliomas) OR (carcinomatose) OR (carcinomatosis) OR (adenoma) OR (adenomas)))

- - **Distinguished by scopus**

TITLE-ABS-KEY ( ( ( mir-212 ) OR ( mir-212 ) OR ( mirna-212 ) OR ( microrna-212 ) ) AND ( ( neoplasms ) OR ( neoplasia ) OR ( neoplasias ) OR ( neoplasm ) OR ( tumors ) OR ( tumor ) OR ( cancer ) OR ( cancers ) OR ( malignancy ) OR ( malignancies ) OR ( malignant AND neoplasms ) OR ( malignant AND neoplasm ) OR ( neoplasm, AND malignant ) OR ( neoplasms, AND malignant ) OR ( benign AND neoplasms ) OR ( neoplasms, AND benign ) OR ( benign AND neoplasm ) OR ( neoplasm, AND benign ) OR ( carcinoma ) OR ( carcinomas ) OR ( adenocarcinoma ) OR ( adenocarcinomas ) OR ( epithelioma ) OR ( epitheliomas ) OR ( carcinomatose ) OR ( carcinomatosis ) OR ( adenoma ) OR ( adenomas ) ) )

- **Web of Science**
  - **Searched by the authors**

TS= (((mir-212) OR (miR-212) OR (miRNA-212) OR (microRNA-212)) AND ((Neoplasms) OR (Neoplasia) OR (Neoplasias) OR (Neoplasm) OR (Tumors) OR (Tumor) OR (Cancer) OR (Cancers) OR (Malignancy) OR (Malignancies) OR (Malignant Neoplasms) OR (Malignant Neoplasm) OR (Neoplasm, Malignant) OR (Neoplasms, Malignant) OR (Benign Neoplasms) OR (Neoplasms, Benign) OR (Benign Neoplasm) OR (Neoplasm, Benign) OR (Carcinoma) OR (Carcinomas) OR (Adenocarcinoma) OR (Adenocarcinomas) OR (Epithelioma) OR (epitheliomas) OR (carcinomatose) OR (carcinomatosis) OR (adenoma) OR (adenomas)))

- - **Distinguished by Web of Science**

TS= (((mir-212)  OR (miR-212)  OR (miRNA-212)  OR (microRNA-212))  AND ((Neoplasms)  OR (Neoplasia)  OR (Neoplasias)  OR (Neoplasm)  OR (Tumors)  OR (Tumor)  OR (Cancer)  OR (Cancers)  OR (Malignancy)  OR (Malignancies)  OR (Malignant Neoplasms)  OR (Malignant Neoplasm)  OR (Neoplasm, Malignant)  OR (Neoplasms, Malignant)  OR (Benign Neoplasms)  OR (Neoplasms, Benign)  OR (Benign Neoplasm)  OR (Neoplasm, Benign)  OR (Carcinoma)  OR (Carcinomas)  OR (Adenocarcinoma)  OR (Adenocarcinomas)  OR (Epithelioma)  OR (epitheliomas)  OR (carcinomatose)  OR (carcinomatosis)  OR (adenoma)  OR (adenomas)))

**Timespan:** All years. **Indexes:** SCI-EXPANDED, SSCI, A&HCI, CPCI-S, CPCI-SSH, BKCI-S, BKCI-SSH, ESCI, CCR-EXPANDED, IC.

- **Embase**
  - **Searched by the authors**

(((mir-212) OR (miR-212) OR (miRNA-212) OR (microRNA-212)) AND ((Neoplasms) OR (Neoplasia) OR (Neoplasias) OR (Neoplasm) OR (Tumors) OR (Tumor) OR (Cancer) OR (Cancers) OR (Malignancy) OR (Malignancies) OR (Malignant Neoplasms) OR (Malignant Neoplasm) OR (Neoplasm, Malignant) OR (Neoplasms, Malignant) OR (Benign Neoplasms) OR (Neoplasms, Benign) OR (Benign Neoplasm) OR (Neoplasm, Benign) OR (Carcinoma) OR (Carcinomas) OR (Adenocarcinoma) OR (Adenocarcinomas) OR (Epithelioma) OR (epitheliomas) OR (carcinomatose) OR (carcinomatosis) OR (adenoma) OR (adenomas))):ti,ab,kw

- - **Distinguished by Embase**

('mir 212':ti,ab,kw OR 'mirna 212':ti,ab,kw OR 'microrna 212':ti,ab,kw) AND (neoplasms:ti,ab,kw OR neoplasia:ti,ab,kw OR neoplasias:ti,ab,kw OR neoplasm:ti,ab,kw OR tumors:ti,ab,kw OR tumor:ti,ab,kw OR cancer:ti,ab,kw OR cancers:ti,ab,kw OR malignancy:ti,ab,kw OR malignancies:ti,ab,kw OR (malignant:ti,ab,kw AND neoplasms:ti,ab,kw) OR (malignant:ti,ab,kw AND neoplasm:ti,ab,kw) OR (neoplasm,:ti,ab,kw AND malignant:ti,ab,kw) OR (neoplasms,:ti,ab,kw AND malignant:ti,ab,kw) OR (benign:ti,ab,kw AND neoplasms:ti,ab,kw) OR (neoplasms,:ti,ab,kw AND benign:ti,ab,kw) OR (benign:ti,ab,kw AND neoplasm:ti,ab,kw) OR (neoplasm,:ti,ab,kw AND benign:ti,ab,kw) OR carcinoma:ti,ab,kw OR carcinomas:ti,ab,kw OR adenocarcinoma:ti,ab,kw OR adenocarcinomas:ti,ab,kw OR epithelioma:ti,ab,kw OR epitheliomas:ti,ab,kw OR carcinomatose:ti,ab,kw OR carcinomatosis:ti,ab,kw OR adenoma:ti,ab,kw OR adenomas:ti,ab,kw)

- **ScienceDirect**
  - **Searched by the authors**

In the field of “Title, abstract or author-specified keywords”:

((mir-212) OR (miR-212) OR (miRNA-212) OR (microRNA-212)) AND ((Neoplasm) OR (Neoplasia) OR (Cancer) OR (Tumor))

- - **Distinguished by ScienceDirect**

Title, abstract, keywords: ((mir-212) OR (miR-212) OR (miRNA-212) OR (microRNA-212)) AND ((Neoplasm) OR (Neoplasia) OR (Cancer) OR (Tumor))
