# Supplementary material for: MiR-212 value in prognosis and diagnosis of cancer and its association with patient characteristics: a systematic review and meta-analysis
Source: Cancer Cell Int. 2022 Apr 26;22:163. doi: 10.1186/s12935-022-02584-0 (PMC9044851; doi:10.1186/s12935-022-02584-0)
Supplement: Supplementary file 2 — Additional file 2: Table S1, S2 and Figure S1–S43. [file 12935_2022_2584_MOESM2_ESM.pdf]

Table S1- Targeted genes and hazard ratios extracted from included articles in prognostic and association evaluation study.

Study ID	Targeted gene	Related functions	Overall Survival	Disease-free survival	Recurrence-free survival	Adjusted Overall Survival	Overall Survival Adjusted for	Disease-free survival Multivariate	Disease-free survival Adjusted for
Meng, X. 2013	MnSoD	Growth, migration, invasion, tumor suppressor	2.91 (1.43-5.78)	3.17 (1.83-5.47)		2.48 (1.21-5.13)	Tumor size (≤5 vs >5 cm), CEA (≤5 vs >5 ng/mL), Histologic grade (I/II vs III)		
Qi, B. 2014	-	Oncogene	2.11 (1.06-4.18)						
Dou, C. 2015	FOXA1	clinical biomarker, cell viability, proliferation	2.26 (1.37-3.71)	2.7 (1.69-4.31)		1.67 (1.01-2.75)	FOXA1, TNM tumor stage, Edmondson grading, tumor size	1.65 (1.01-2.7)	FOXA1, TNM tumor stage, Edmondson grading, tumor size
Li, D. 2015	PXN	Migration, invasion, tumor suppressor	2.16 (1.21-3.85)						
Tu, H. 2015	FOXA1	tumor growth	2.01 (1.16-3.48)	3.55 (1.64-7.65)		2.15 (1.26-3.68)	Serum AFP level, Tumor size, Edmondson-S teiner grading, TNM tumor stage	3.55 (1.64-7.65)	Serum AFP level, Tumor size, Edmondson-S teiner grading, TNM tumor stage
Gu, C. 2017	XIAP	Tumor progression, proliferation, migration, invasion, prognostic predictor	2.07 (0.99-4.34)		2.03 (1.00-4.12)				
Jiang, C. 2017	SOX4	Migration, invasion, tumor suppressor	2.83 (1.17-6.84)	2.73 (1.24-6)					
Lu, Z. 2017	prrx2	prognostic marker, tumor development, vascular invasion, metastasis	2.07 (0.97-4.39)	2.17 (1.09-4.31)		2.91 (1.13-7.58)	Age, Menopausal status, Tumor size, Lymph node status, Histological grade		
Tang, T.T. 2017	SOX4	cell migration, invasion, tumor suppressor	2.34 (1.48-3.70)						
Wu, Z. 2017	-	Oncogene	0.38 (0.18-0.79)						
Zhou, Y. 2017	EN-2	PC progression							
Qu, H. 2018	BMI1	cancer development	2.88 (1.60-5.18)						
Tong, Z. 2018	FOXA1	cell proliferation, migration, invasion, apoptosis, clinical biomarker							
Wang, F. 2018			2.52 (1.34-4.74)						
Chen, J. 2019	CTGF	Tumor encapsulation, vascular invaison	2.13 (1.24-3.67)		1.76 (1.09-2.84)				
Mou, T. 2019	SOX4	Tumor suppressor, invasion, ,LN metastasis	2.59 (1.10-6.08)						
Yue, H. 2019	HIF-1a	Oncogene	0.44 (0.21-0.94)						
Azar, M. 2020	nothing	Tumor suppressor							

Kang, Y. 2020	ELF3	cell proliferation				
Shao, J. 2020	SOX4	potential circulating biomarker, proliferation	4.56 (2.95-7.07)			
Yuan, Z. 2020	FOXA1	tumor suppressor	1.93 (1.14-3.28)			
Zhang, L. 2020	MAP3K3	tumor supressor	4.85 (1.64-14.29)		6.62 (0.82-52.63)	FIGO stage (I-II vs III-IV)

Table S2- Patient properties and sensitivity and specificity extracted from the included articles of the diagnostic evaluation study.

Study ID	Age	Gender (PM/PF|CM/CF)	No. (patient/control)	Article Reported		Youden Index		Index of Union	
				Sensitivity (%)	Specificity (%)	Sensitivity (%)	Specificity (%)	Sensitivity (%)	Specificity (%)
Miah, S. 2012	71/58	NR	62/50	54.2	64	-	-	-	-
Cote, G. 2014	66.5/ 65.1	24/16| 30/25	40/54	90	83	90	83.33	90	83.33
			25/45	96	100	96	100	96	100
Ramalinga, M. 2015	NR	NR	40/32	-	-	82.5	53.13	70	59.38
Bagheri, A. 2016	51-73/ 48-71	15/2| 15/2	17/17	45	91	47.06	88.24	70.59	64.71
			17/17	46.7	93.3	-	-	-	-
Damavandi, Z. 2016	NR	NR	31	-	-	35.48	93.55	58.06	58.06
Wang, F. 2018	NR	70/10|NR	80/93	68.75	66.67	68.75	66.67	68.75	66.67
Pu, X. 2020	NR	21/15|NR	36/65	-	-	52.78	81.54	66.67	64.62
Shao, J. 2020	61.5/ 58.5	62/48| 63/37	110/100	95.1	78.7	93.64	89	93.64	89

Abbreviations: PM: number of male patients, PF: number of female patients, CM: number of male controls, CF: number of female controls.

Overall Survival in Association With MiR-212 Expression

Model	Study name	Hazard ratio	Lower limit	Upper limit	Z-Value	p-Value
	Meng, X. 2013	2.907	1.448	5.835	3.002	0.003
	Qi, B. 2014	2.106	1.062	4.178	2.132	0.033
	Dou, C. 2015	2.257	1.373	3.709	3.211	0.001
	Li, D. 2015	2.156	1.208	3.848	2.601	0.009
	Tu, H. 2015	2.011	1.162	3.478	2.497	0.013
	Gu, C. 2017	2.072	0.989	4.341	1.930	0.054
	Jiang, C. 2017	2.831	1.172	6.837	2.313	0.021
	Lu, Z. 2017	2.066	0.974	4.383	1.891	0.059
	Tang, T.T. 2017	2.342	1.481	3.703	3.639	0.000
	Wu, Z. 2017	0.380	0.183	0.792	-2.584	0.010
	Qu, H. 2018	2.876	1.597	5.177	3.521	0.000
	Wang, F. 2018	2.520	1.340	4.739	2.870	0.004
	Chen, J. 2019	2.131	1.238	3.670	2.729	0.006
	Mou, T. 2019	2.590	1.104	6.076	2.187	0.029
	Yue, H. 2019	0.440	0.207	0.939	-2.124	0.034
	Shao, J. 2020	4.564	2.945	7.074	6.792	0.000
	Yuan, Z. 2020	1.929	1.136	3.278	2.430	0.015
	Zhang, L. 2020	4.854	1.643	14.342	2.858	0.004
Fixed		2.190	1.895	2.532	10.601	0.000
Random		2.081	1.593	2.717	5.382	0.000

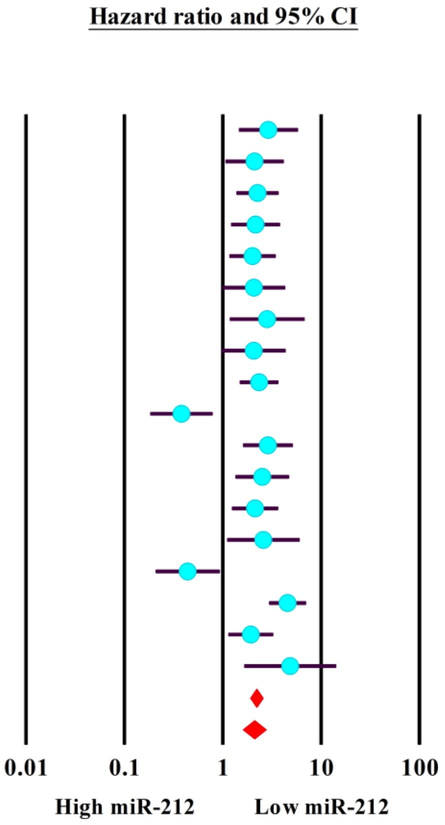

Figure S1- Forrest plot of overall survival (OS) in association with low miR-212 expression.

Sensitivity Analysis of Overall Survival in Association With MiR-212 Expression

Study name	Statistics with study removed				
	Point	Lower limit	Upper limit	Z-Value	p-Value
Meng, X. 2013	2.041	1.543	2.699	4.998	0.000
Qi, B. 2014	2.078	1.568	2.754	5.090	0.000
Dou, C. 2015	2.067	1.551	2.755	4.951	0.000
Li, D. 2015	2.074	1.561	2.757	5.027	0.000
Tu, H. 2015	2.083	1.566	2.771	5.045	0.000
Gu, C. 2017	2.080	1.571	2.753	5.118	0.000
Jiang, C. 2017	2.050	1.554	2.706	5.073	0.000
Lu, Z. 2017	2.080	1.572	2.753	5.123	0.000
Tang, T.T. 2017	2.061	1.543	2.752	4.903	0.000
Wu, Z. 2017	2.293	1.850	2.842	7.577	0.000
Qu, H. 2018	2.037	1.537	2.701	4.947	0.000
Wang, F. 2018	2.055	1.550	2.726	4.999	0.000
Chen, J. 2019	2.075	1.560	2.761	5.011	0.000
Mou, T. 2019	2.058	1.558	2.718	5.083	0.000
Yue, H. 2019	2.265	1.799	2.851	6.959	0.000
Shao, J. 2020	1.965	1.520	2.540	5.159	0.000
Yuan, Z. 2020	2.089	1.570	2.780	5.058	0.000
Zhang, L. 2020	2.018	1.539	2.645	5.082	0.000
	2.081	1.593	2.717	5.382	0.000

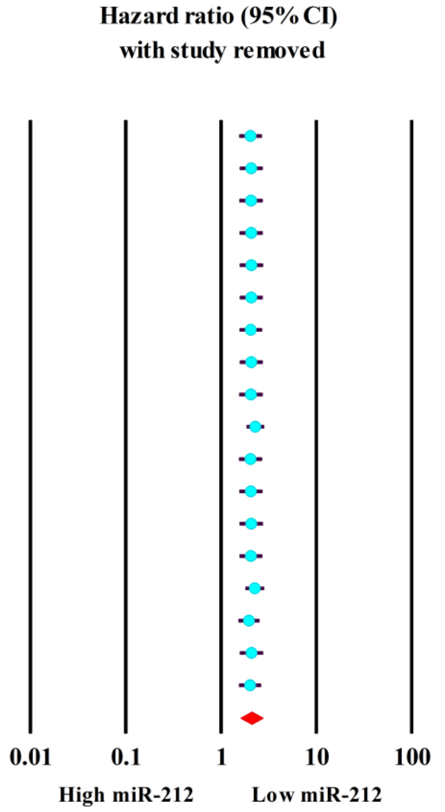

Figure S2- Sensitivity analysis of overall survival (OS) in association with low miR-212 expression using the one-study-removed method.

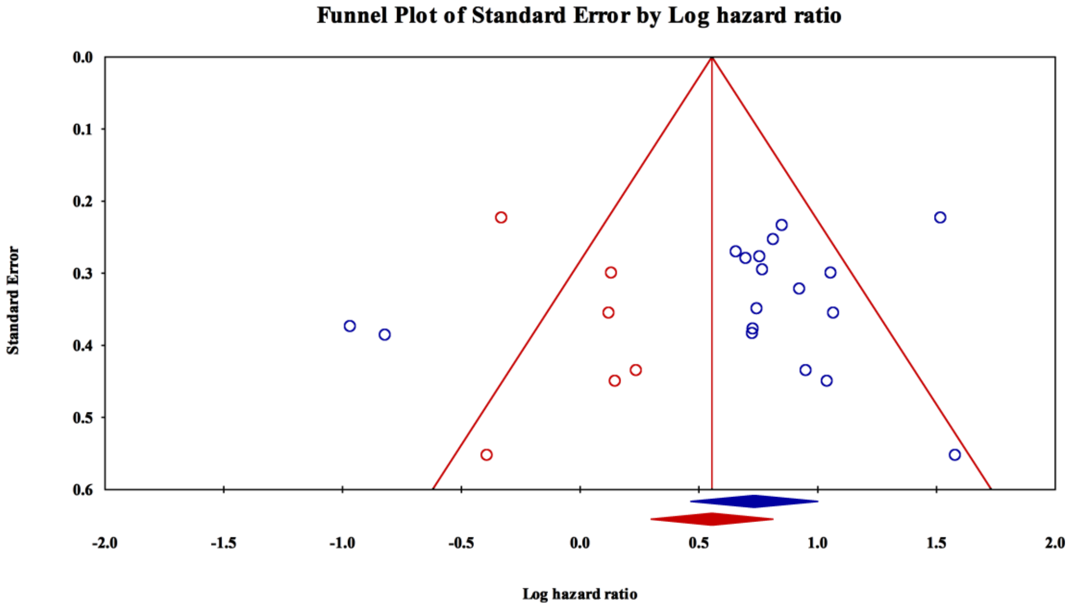

Figure S3- Begg's funnel plot of studies included in the meta-analysis of overall survival (OS) in association with low miR-212 expression.

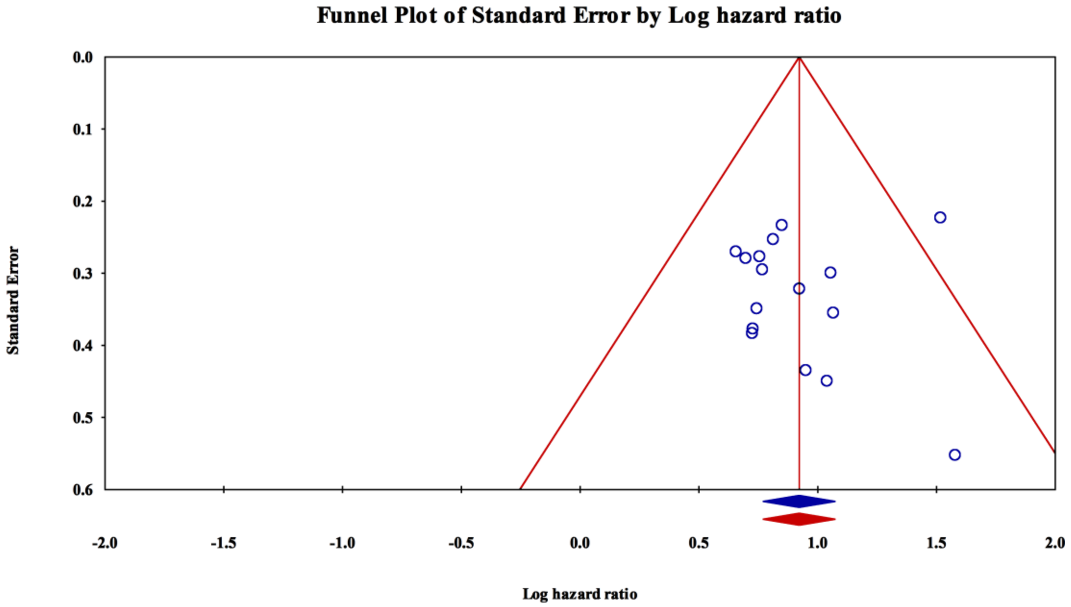

Figure S4- Begg's funnel plot of studies included in the meta-analysis of overall survival (OS) in association with low miR-212 expression after omitting studies on pancreatic ductal adenocarcinoma (PDAC).

Overall Survival in Association With MiR-212 Expression Sub-Grouped by Publication Year

Model	Group by Publication year	Study name	Hazard ratio	Lower limit	Upper limit	Z-Value	p-Value
	<2017	Meng, X. 2013	2.907	1.448	5.835	3.002	0.003
	<2017	Qi, B. 2014	2.106	1.062	4.178	2.132	0.033
	<2017	Dou, C. 2015	2.257	1.373	3.709	3.211	0.001
	<2017	Li, D. 2015	2.156	1.208	3.848	2.601	0.009
	<2017	Tu, H. 2015	2.011	1.162	3.478	2.497	0.013
Fixed	<2017		2.234	1.719	2.904	6.011	0.000
Random	<2017		2.234	1.719	2.904	6.011	0.000
	>2017	Qu, H. 2018	2.876	1.597	5.177	3.521	0.000
	>2017	Wang, F. 2018	2.520	1.340	4.739	2.870	0.004
	>2017	Chen, J. 2019	2.131	1.238	3.670	2.729	0.006
	>2017	Mou, T. 2019	2.590	1.104	6.076	2.187	0.029
	>2017	Yue, H. 2019	0.440	0.207	0.939	-2.124	0.034
	>2017	Shao, J. 2020	4.564	2.945	7.074	6.792	0.000
	>2017	Yuan, Z. 2020	1.929	1.136	3.278	2.430	0.015
	>2017	Zhang, L. 2020	4.854	1.643	14.342	2.858	0.004
Fixed	>2017		2.463	1.986	3.055	8.197	0.000
Random	>2017		2.284	1.433	3.641	3.472	0.001
	2017.00	Gu, C. 2017	2.072	0.989	4.341	1.930	0.054
	2017.00	Jiang, C. 2017	2.831	1.172	6.837	2.313	0.021
	2017.00	Lu, Z. 2017	2.066	0.974	4.383	1.891	0.059
	2017.00	Tang, T.T. 2017	2.342	1.481	3.703	3.639	0.000
	2017.00	Wu, Z. 2017	0.380	0.183	0.792	-2.584	0.010
Fixed	2017.00		1.715	1.277	2.303	3.586	0.000
Random	2017.00		1.611	0.811	3.200	1.361	0.174
Fixed	Overall		2.190	1.895	2.532	10.601	0.000
Random	Overall		2.173	1.749	2.699	7.015	0.000

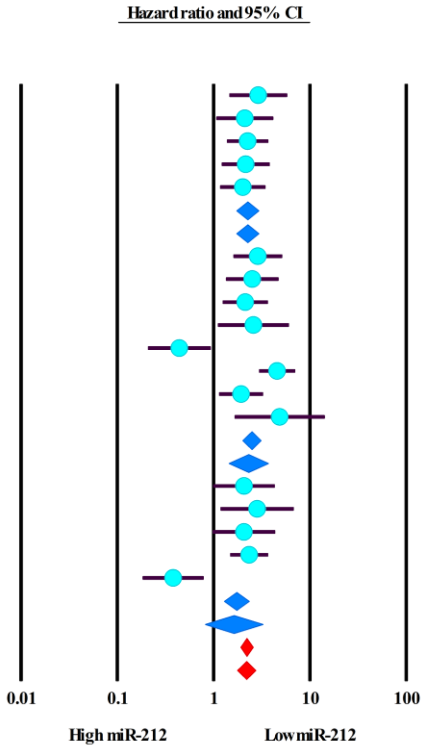

Figure S5- Forrest plot of overall survival (OS) in association with low miR-212 expression: sub-grouped by publication year.

Overall Survival in Association With MiR-212 Expression Sub-Grouped by Sample Size

Model	Group by Sample size	Study name	Hazard ratio	Lower limit	Upper limit	Z-Value	p-Value
	<75	Qi, B. 2014	2.106	1.062	4.178	2.132	0.033
	<75	Li, D. 2015	2.156	1.208	3.848	2.601	0.009
	<75	Gu, C. 2017	2.072	0.989	4.341	1.930	0.054
	<75	Jiang, C. 2017	2.831	1.172	6.837	2.313	0.021
	<75	Wu, Z. 2017	0.380	0.183	0.792	-2.584	0.010
	<75	Qu, H. 2018	2.876	1.597	5.177	3.521	0.000
	<75	Mou, T. 2019	2.590	1.104	6.076	2.187	0.029
	<75	Yue, H. 2019	0.440	0.207	0.939	-2.124	0.034
	<75	Zhang, L. 2020	4.854	1.643	14.342	2.858	0.004
Fixed	<75		1.703	1.336	2.171	4.297	0.000
Random	<75		1.722	1.002	2.961	1.966	0.049
	>75	Meng, X. 2013	2.907	1.448	5.835	3.002	0.003
	>75	Dou, C. 2015	2.257	1.373	3.709	3.211	0.001
	>75	Tu, H. 2015	2.011	1.162	3.478	2.497	0.013
	>75	Lu, Z. 2017	2.066	0.974	4.383	1.891	0.059
	>75	Tang, T.T. 2017	2.342	1.481	3.703	3.639	0.000
	>75	Wang, F. 2018	2.520	1.340	4.739	2.870	0.004
	>75	Chen, J. 2019	2.131	1.238	3.670	2.729	0.006
	>75	Shao, J. 2020	4.564	2.945	7.074	6.792	0.000
	>75	Yuan, Z. 2020	1.929	1.136	3.278	2.430	0.015
Fixed	>75		2.518	2.102	3.016	10.016	0.000
Random	>75		2.498	2.042	3.055	8.904	0.000
Fixed	Overall		2.190	1.895	2.532	10.601	0.000
Random	Overall		2.387	1.976	2.884	9.031	0.000

Hazard ratio and 95% CI

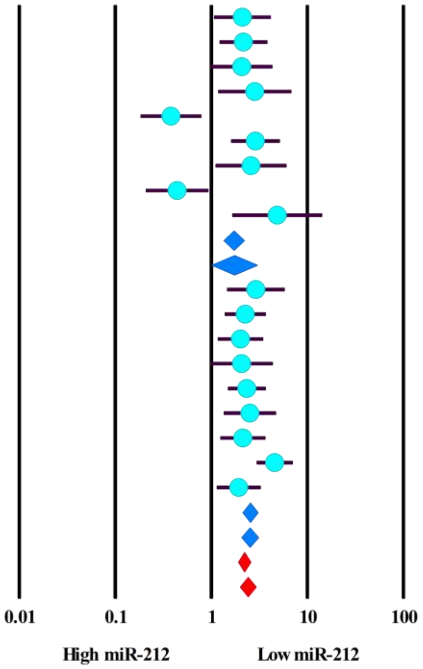

Figure S6- Forrest plot of overall survival (OS) in association with low miR-212 expression: sub-grouped by sample size.

Overall Survival in Association With MiR-212 Expression Sub-Grouped by Cancer Type

Model	Group by Cancer type	Study name	Hazard ratio	Lower limit	Upper limit	Z-Value	p-Value
	CRC	Meng, X. 2013	2.907	1.448	5.835	3.002	0.003
	CRC	Gu, C. 2017	2.072	0.989	4.341	1.930	0.054
	CRC	Mou, T. 2019	2.590	1.104	6.076	2.187	0.029
Fixed	CRC		2.508	1.622	3.878	4.133	0.000
Random	CRC		2.508	1.622	3.878	4.133	0.000
	Gastric	Li, D. 2015	2.156	1.208	3.848	2.601	0.009
	Gastric	Shao, J. 2020	4.564	2.945	7.074	6.792	0.000
Fixed	Gastric		3.474	2.450	4.927	6.985	0.000
Random	Gastric		3.216	1.545	6.696	3.123	0.002
	HCC	Dou, C. 2015	2.257	1.373	3.709	3.211	0.001
	HCC	Tu, H. 2015	2.011	1.162	3.478	2.497	0.013
	HCC	Wang, F. 2018	2.520	1.340	4.739	2.870	0.004
	HCC	Chen, J. 2019	2.131	1.238	3.670	2.729	0.006
	HCC	Yuan, Z. 2020	1.929	1.136	3.278	2.430	0.015
Fixed	HCC		2.145	1.681	2.736	6.135	0.000
Random	HCC		2.145	1.681	2.736	6.135	0.000
	PDAC	Wu, Z. 2017	0.380	0.183	0.792	-2.584	0.010
	PDAC	Yue, H. 2019	0.440	0.207	0.939	-2.124	0.034
Fixed	PDAC		0.408	0.241	0.691	-3.334	0.001
Random	PDAC		0.408	0.241	0.691	-3.334	0.001
Fixed	Overall		2.069	1.743	2.457	8.298	0.000
Random	Overall		1.828	1.511	2.211	6.205	0.000

Hazard ratio and 95% CI

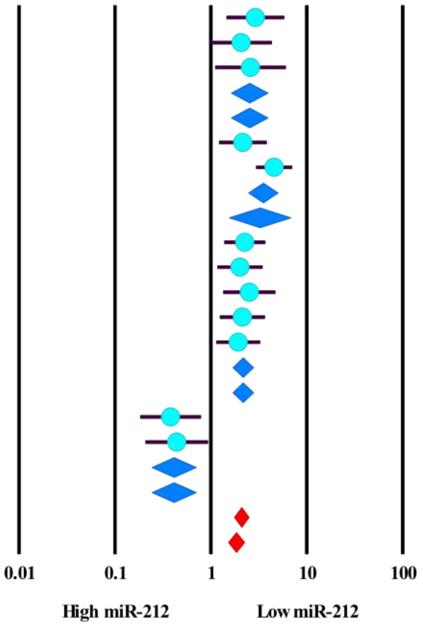

Figure S7- Forrest plot of overall survival (OS) in association with low miR-212 expression: sub-grouped by cancer type.

Overall Survival in Association With MiR-212 Expression Sub-Grouped by Cancer Type (PDAC, Others)

Model	Group by Cancer type	Study name	Hazard ratio	Lower limit	Upper limit	Z-Value	p-Value
	others	Meng, X. 2013	2.907	1.448	5.835	3.002	0.003
	others	Qi, B. 2014	2.106	1.062	4.178	2.132	0.033
	others	Dou, C. 2015	2.257	1.373	3.709	3.211	0.001
	others	Li, D. 2015	2.156	1.208	3.848	2.601	0.009
	others	Tu, H. 2015	2.011	1.162	3.478	2.497	0.013
	others	Gu, C. 2017	2.072	0.989	4.341	1.930	0.054
	others	Jiang, C. 2017	2.831	1.172	6.837	2.313	0.021
	others	Lu, Z. 2017	2.066	0.974	4.383	1.891	0.059
	others	Tang, T.T. 2017	2.342	1.481	3.703	3.639	0.000
	others	Qu, H. 2018	2.876	1.597	5.177	3.521	0.000
	others	Wang, F. 2018	2.520	1.340	4.739	2.870	0.004
	others	Chen, J. 2019	2.131	1.238	3.670	2.729	0.006
	others	Mou, T. 2019	2.590	1.104	6.076	2.187	0.029
	others	Shao, J. 2020	4.564	2.945	7.074	6.792	0.000
	others	Yuan, Z. 2020	1.929	1.136	3.278	2.430	0.015
	others	Zhang, L. 2020	4.854	1.643	14.342	2.858	0.004
Fixed	others		2.514	2.162	2.923	11.982	0.000
Random	others		2.514	2.162	2.923	11.982	0.000
	PDAC	Wu, Z. 2017	0.380	0.183	0.792	-2.584	0.010
	PDAC	Yue, H. 2019	0.440	0.207	0.939	-2.124	0.034
Fixed	PDAC		0.408	0.241	0.691	-3.334	0.001
Random	PDAC		0.408	0.241	0.691	-3.334	0.001
Fixed	Overall		2.190	1.895	2.532	10.601	0.000
Random	Overall		2.190	1.895	2.532	10.601	0.000

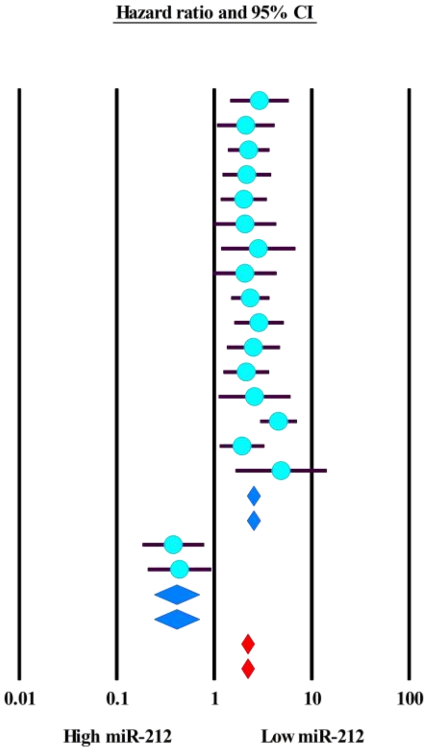

Figure S8- Forrest plot of overall survival (OS) in association with low miR-212 expression: sub-grouped by cancer type (PDAC vs. others).

Overall Survival in Association With MiR-212 Expression Sub-Grouped by Stage

Model	Group by Stage	Study name	Hazard ratio	Lower limit	Upper limit	Z-Value	p-Value
	I-IV	Dou, C. 2015	2.257	1.373	3.709	3.211	0.001
	I-IV	Tu, H. 2015	2.011	1.162	3.478	2.497	0.013
	I-IV	Gu, C. 2017	2.072	0.989	4.341	1.930	0.054
	I-IV	Jiang, C. 2017	2.831	1.172	6.837	2.313	0.021
	I-IV	Tang, T.T. 2017	2.342	1.481	3.703	3.639	0.000
	I-IV	Wu, Z. 2017	0.380	0.183	0.792	-2.584	0.010
	I-IV	Wang, F. 2018	2.520	1.340	4.739	2.870	0.004
	I-IV	Mou, T. 2019	2.590	1.104	6.076	2.187	0.029
	I-IV	Yue, H. 2019	0.440	0.207	0.939	-2.124	0.034
	I-IV	Shao, J. 2020	4.564	2.945	7.074	6.792	0.000
	I-IV	Yuan, Z. 2020	1.929	1.136	3.278	2.430	0.015
	I-IV	Zhang, L. 2020	4.854	1.643	14.342	2.858	0.004
Fixed	I-IV		2.121	1.779	2.528	8.383	0.000
Random	I-IV		1.944	1.304	2.898	3.265	0.001
	others	Meng, X. 2013	2.907	1.448	5.835	3.002	0.003
	others	Qi, B. 2014	2.106	1.062	4.178	2.132	0.033
	others	Li, D. 2015	2.156	1.208	3.848	2.601	0.009
	others	Lu, Z. 2017	2.066	0.974	4.383	1.891	0.059
	others	Qu, H. 2018	2.876	1.597	5.177	3.521	0.000
	others	Chen, J. 2019	2.131	1.238	3.670	2.729	0.006
Fixed	others		2.346	1.816	3.031	6.520	0.000
Random	others		2.346	1.816	3.031	6.520	0.000
Fixed	Overall		2.190	1.895	2.532	10.601	0.000
Random	Overall		2.221	1.790	2.755	7.250	0.000

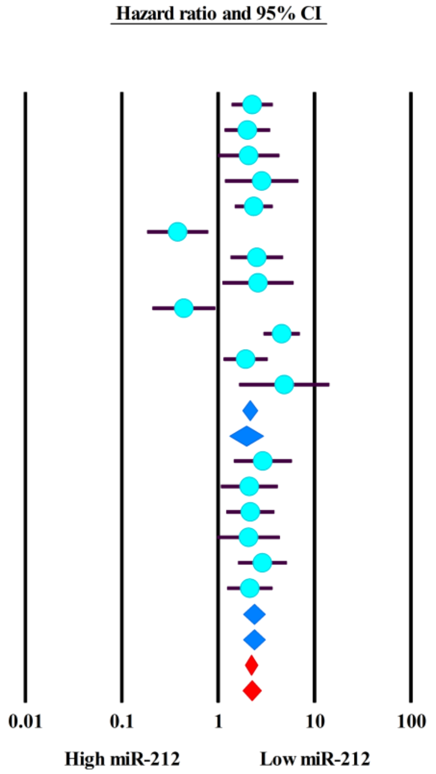

Figure S9- Forrest plot of overall survival (OS) in association with low miR-212 expression: sub-grouped by stage.

Overall Survival in Association With MiR-212 Expression Sub-Grouped by Follow-up Period

Model	Group by Follow-up period	Study name	Hazard ratio	Lower limit	Upper limit	Z-Value	p-Value
	<=60	Dou, C. 2015	2.257	1.373	3.709	3.211	0.001
	<=60	Tu, H. 2015	2.011	1.162	3.478	2.497	0.013
	<=60	Gu, C. 2017	2.072	0.989	4.341	1.930	0.054
	<=60	Jiang, C. 2017	2.831	1.172	6.837	2.313	0.021
	<=60	Tang, T.T. 2017	2.342	1.481	3.703	3.639	0.000
	<=60	Wu, Z. 2017	0.380	0.183	0.792	-2.584	0.010
	<=60	Wang, F. 2018	2.520	1.340	4.739	2.870	0.004
	<=60	Mou, T. 2019	2.590	1.104	6.076	2.187	0.029
	<=60	Yue, H. 2019	0.440	0.207	0.939	-2.124	0.034
	<=60	Shao, J. 2020	4.564	2.945	7.074	6.792	0.000
	<=60	Yuan, Z. 2020	1.929	1.136	3.278	2.430	0.015
Fixed	<=60		2.074	1.735	2.478	8.025	0.000
Random	<=60		1.833	1.214	2.767	2.882	0.004
	>60	Meng, X. 2013	2.907	1.448	5.835	3.002	0.003
	>60	Qi, B. 2014	2.106	1.062	4.178	2.132	0.033
	>60	Li, D. 2015	2.156	1.208	3.848	2.601	0.009
	>60	Lu, Z. 2017	2.066	0.974	4.383	1.891	0.059
	>60	Qu, H. 2018	2.876	1.597	5.177	3.521	0.000
	>60	Chen, J. 2019	2.131	1.238	3.670	2.729	0.006
	>60	Zhang, L. 2020	4.854	1.643	14.342	2.858	0.004
Fixed	>60		2.438	1.900	3.129	7.003	0.000
Random	>60		2.438	1.900	3.129	7.003	0.000
Fixed	Overall		2.190	1.895	2.532	10.601	0.000
Random	Overall		2.258	1.824	2.796	7.483	0.000

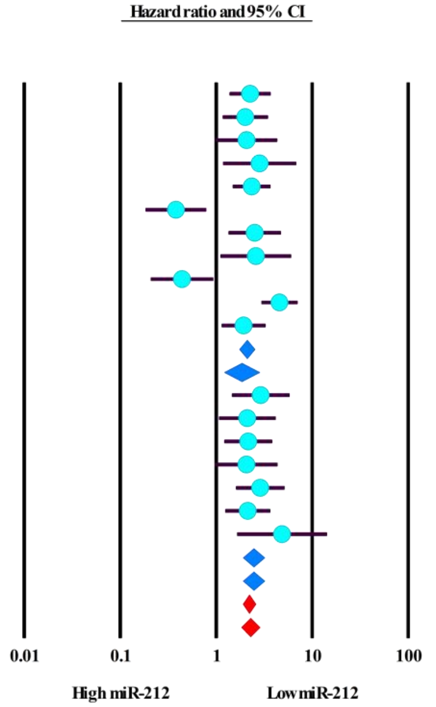

Figure S10- Forrest plot of overall survival (OS) in association with low miR-212 expression: sub-grouped by follow-up period.

Overall Survival in Association With MiR-212 Expression Sub-Grouped by Cut-off Point

Model	Group by Cut-off determination	Study name	Hazard ratio	Lower limit	Upper limit	Z-Value	p-Value
	median	Meng, X. 2013	2.907	1.448	5.835	3.002	0.003
	median	Qi, B. 2014	2.106	1.062	4.178	2.132	0.033
	median	Li, D. 2015	2.156	1.208	3.848	2.601	0.009
	median	Tu, H. 2015	2.011	1.162	3.478	2.497	0.013
	median	Jiang, C. 2017	2.831	1.172	6.837	2.313	0.021
	median	Tang, T.T. 2017	2.342	1.481	3.703	3.639	0.000
	median	Wu, Z. 2017	0.380	0.183	0.792	-2.584	0.010
	median	Qu, H. 2018	2.876	1.597	5.177	3.521	0.000
	median	Wang, F. 2018	2.520	1.340	4.739	2.870	0.004
	median	Chen, J. 2019	2.131	1.238	3.670	2.729	0.006
	median	Mou, T. 2019	2.590	1.104	6.076	2.187	0.029
	median	Shao, J. 2020	4.564	2.945	7.074	6.792	0.000
	median	Zhang, L. 2020	4.854	1.643	14.342	2.858	0.004
Fixed	median		2.412	2.035	2.859	10.156	0.000
Random	median		2.320	1.713	3.141	5.440	0.000
	others	Dou, C. 2015	2.257	1.373	3.709	3.211	0.001
	others	Gu, C. 2017	2.072	0.989	4.341	1.930	0.054
	others	Lu, Z. 2017	2.066	0.974	4.383	1.891	0.059
	others	Yue, H. 2019	0.440	0.207	0.939	-2.124	0.034
	others	Yuan, Z. 2020	1.929	1.136	3.278	2.430	0.015
Fixed	others		1.693	1.282	2.235	3.713	0.000
Random	others		1.568	0.915	2.690	1.635	0.102
Fixed	Overall		2.190	1.895	2.532	10.601	0.000
Random	Overall		2.112	1.621	2.750	5.543	0.000

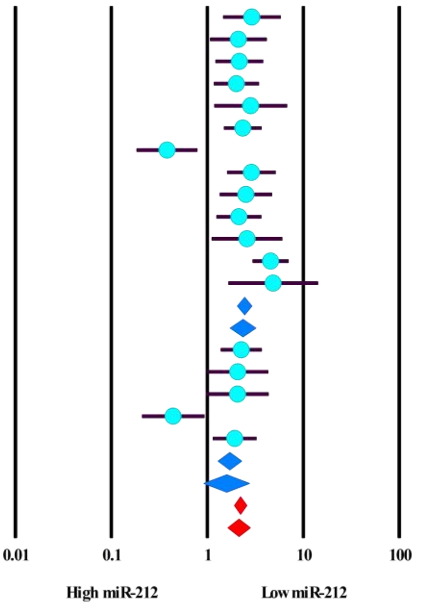

Figure S11- Forrest plot of overall survival (OS) in association with low miR-212 expression: sub-grouped by cut-off determination method.

Overall Survival in Association With MiR-212 Expression Sub-Grouped by Specimen

Model	Group by Specimen	Study name	Hazard ratio	Lower limit	Upper limit	Z-Value	p-Value
	serum	Wang, F. 2018	2.520	1.340	4.739	2.870	0.004
	serum	Shao, J. 2020	4.564	2.945	7.074	6.792	0.000
Fixed	serum		3.763	2.626	5.393	7.216	0.000
Random	serum		3.549	1.996	6.308	4.316	0.000
	tissue	Meng, X. 2013	2.907	1.448	5.835	3.002	0.003
	tissue	Qi, B. 2014	2.106	1.062	4.178	2.132	0.033
	tissue	Dou, C. 2015	2.257	1.373	3.709	3.211	0.001
	tissue	Li, D. 2015	2.156	1.208	3.848	2.601	0.009
	tissue	Tu, H. 2015	2.011	1.162	3.478	2.497	0.013
	tissue	Gu, C. 2017	2.072	0.989	4.341	1.930	0.054
	tissue	Jiang, C. 2017	2.831	1.172	6.837	2.313	0.021
	tissue	Lu, Z. 2017	2.066	0.974	4.383	1.891	0.059
	tissue	Tang, T.T. 2017	2.342	1.481	3.703	3.639	0.000
	tissue	Wu, Z. 2017	0.380	0.183	0.792	-2.584	0.010
	tissue	Qu, H. 2018	2.876	1.597	5.177	3.521	0.000
	tissue	Chen, J. 2019	2.131	1.238	3.670	2.729	0.006
	tissue	Mou, T. 2019	2.590	1.104	6.076	2.187	0.029
	tissue	Yue, H. 2019	0.440	0.207	0.939	-2.124	0.034
	tissue	Yuan, Z. 2020	1.929	1.136	3.278	2.430	0.015
	tissue	Zhang, L. 2020	4.854	1.643	14.342	2.858	0.004
Fixed	tissue		1.973	1.684	2.311	8.407	0.000
Random	tissue		1.933	1.473	2.537	4.750	0.000
Fixed	Overall		2.190	1.895	2.532	10.601	0.000
Random	Overall		2.160	1.689	2.761	6.139	0.000

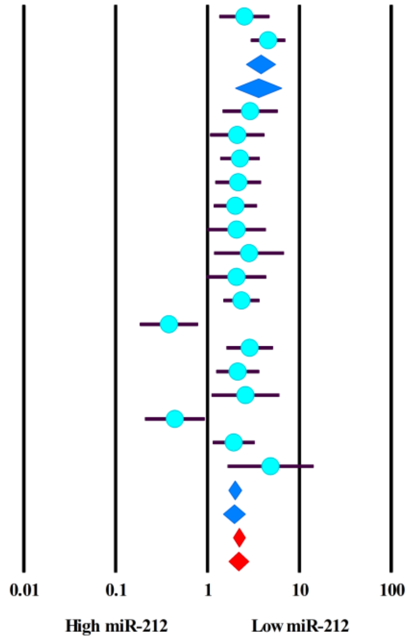

Figure S12- Forrest plot of overall survival (OS) in association with low miR-212 expression: sub-grouped by the specimen.

Overall Survival in Association With MiR-212 Expression Sub-Grouped by MiR-212 Type

Model	Group by MiR-212 type	Study name	Hazard ratio	Lower limit	Upper limit	Z-Value	p-Value
	miR-212	Meng, X. 2013	2.907	1.448	5.835	3.002	0.003
	miR-212	Qi, B. 2014	2.106	1.062	4.178	2.132	0.033
	miR-212	Dou, C. 2015	2.257	1.373	3.709	3.211	0.001
	miR-212	Li, D. 2015	2.156	1.208	3.848	2.601	0.009
	miR-212	Tu, H. 2015	2.011	1.162	3.478	2.497	0.013
	miR-212	Gu, C. 2017	2.072	0.989	4.341	1.930	0.054
	miR-212	Jiang, C. 2017	2.831	1.172	6.837	2.313	0.021
	miR-212	Tang, T.T. 2017	2.342	1.481	3.703	3.639	0.000
	miR-212	Wu, Z. 2017	0.380	0.183	0.792	-2.584	0.010
	miR-212	Qu, H. 2018	2.876	1.597	5.177	3.521	0.000
	miR-212	Wang, F. 2018	2.520	1.340	4.739	2.870	0.004
	miR-212	Mou, T. 2019	2.590	1.104	6.076	2.187	0.029
	miR-212	Yue, H. 2019	0.440	0.207	0.939	-2.124	0.034
	miR-212	Shao, J. 2020	4.564	2.945	7.074	6.792	0.000
Fixed	miR-212		2.189	1.861	2.574	9.466	0.000
Random	miR-212		2.001	1.435	2.791	4.085	0.000
	miR-212-3p	Chen, J. 2019	2.131	1.238	3.670	2.729	0.006
	miR-212-3p	Yuan, Z. 2020	1.929	1.136	3.278	2.430	0.015
	miR-212-3p	Zhang, L. 2020	4.854	1.643	14.342	2.858	0.004
Fixed	miR-212-3p		2.228	1.558	3.188	4.385	0.000
Random	miR-212-3p		2.259	1.525	3.344	4.069	0.000
	miR-212-5p	Lu, Z. 2017	2.066	0.974	4.383	1.891	0.059
Fixed	miR-212-5p		2.066	0.974	4.383	1.891	0.059
Random	miR-212-5p		2.066	0.974	4.383	1.891	0.059
Fixed	Overall		2.190	1.895	2.532	10.601	0.000
Random	Overall		2.101	1.652	2.672	6.051	0.000

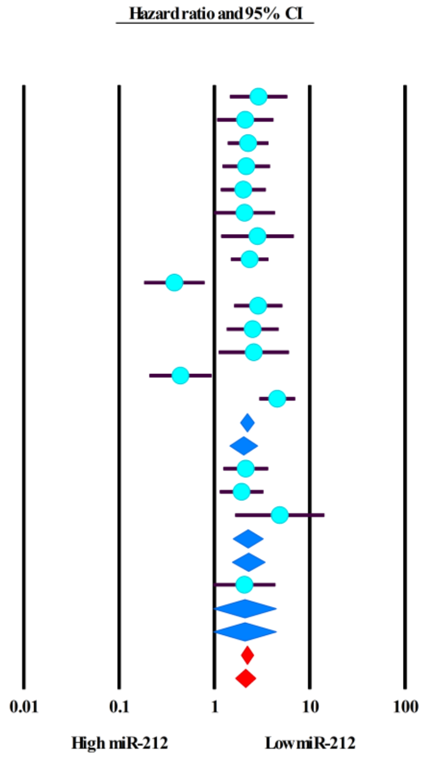

Figure S13- Forrest plot of overall survival (OS) in association with low miR-212 expression: sub-grouped by miR-212 type.

Adjusted Overall Survival in Association With MiR-212 Expression

Model	Study name	Hazard ratio	Lower limit	Upper limit	Z-Value	p-Value
	Meng, X. 2013	2.481	1.203	5.116	2.462	0.014
	Dou, C. 2015	1.665	1.007	2.753	1.986	0.047
	Tu, H. 2015	2.148	1.255	3.677	2.788	0.005
	Lu, Z. 2017	2.907	1.123	7.523	2.200	0.028
	Zhang, L. 2020	6.623	0.828	52.937	1.783	0.075
Fixed		2.121	1.561	2.881	4.811	0.000
Random		2.121	1.561	2.881	4.811	0.000

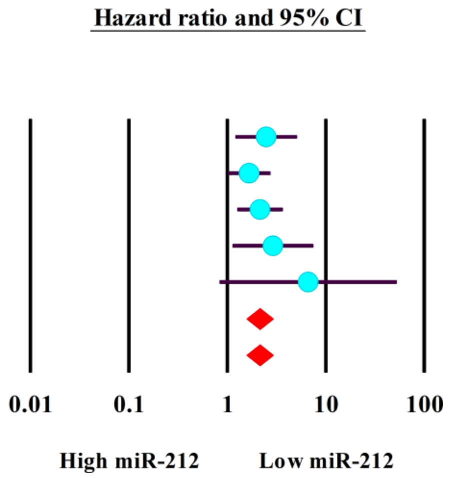

Figure S14- Forrest plot of Adjusted overall survival (OS) in association with low miR-212 expression.

Disease-Free Survival in Association With MiR-212 Expression

Model	Study name	Hazard ratio	Lower limit	Upper limit	Z-Value	p-Value
	Meng, X. 2013	3.166	1.833	5.467	4.135	0.000
	Dou, C. 2015	2.697	1.687	4.314	4.141	0.000
	Tu, H. 2015	3.547	1.644	7.653	3.227	0.001
	Jiang, C. 2017	2.725	1.238	5.999	2.491	0.013
	Lu, Z. 2017	2.167	1.089	4.315	2.201	0.028
Fixed		2.812	2.138	3.700	7.389	0.000
Random		2.812	2.138	3.700	7.389	0.000

(Statistics for each study)

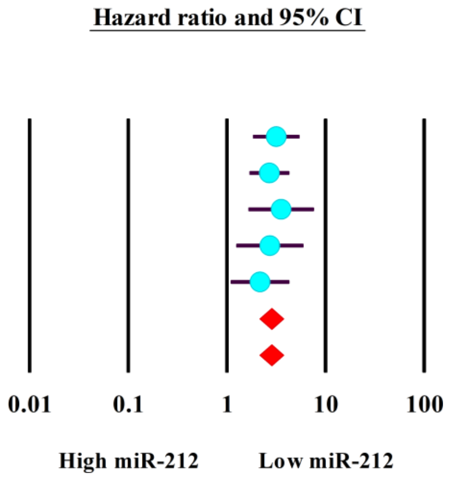

Figure S15- Forrest plot of disease-free survival (DFS) in association with low miR-212 expression.

Adjusted Disease-Free Survival in Association With MiR-212 Expression

Model	Study name	Hazard ratio	Lower limit	Upper limit	Z-Value	p-Value
	Dou, C. 2015	1.648	1.008	2.695	1.991	0.046
	Tu, H. 2015	3.547	1.644	7.653	3.227	0.001
Fixed		2.059	1.360	3.115	3.416	0.001
Random		2.278	1.085	4.786	2.175	0.030

(Statistics for each study)

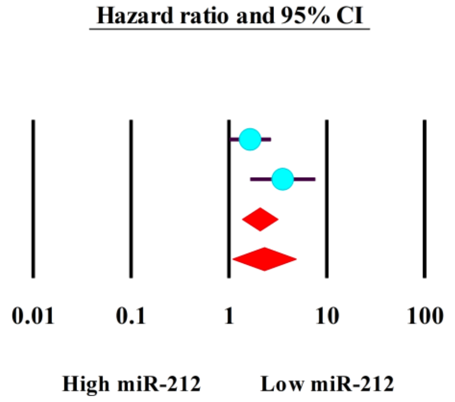

Figure S16- Forrest plot of adjusted disease-free survival (DFS) in association with low miR-212 expression.

Recurrence-Free Survival in Association With MiR-212 Expression

Model	Study name	Hazard ratio	Lower limit	Upper limit	Z-Value	p-Value
	Gu, C. 2017	2.033	1.003	4.121	1.967	0.049
	Chen, J. 2019	1.761	1.092	2.840	2.321	0.020
Fixed		1.842	1.240	2.737	3.025	0.002
Random		1.842	1.240	2.737	3.025	0.002

Statistics for each study

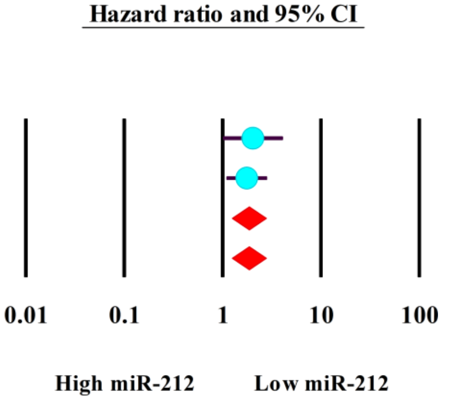

Figure S17- Forrest plot of recurrence-free survival (RFS) in association with low miR-212 expression.

Overall Analysis of Low Mir-212 Expression Association With Gender

Model	Study name	Odds ratio	Lower limit	Upper limit	Z-Value	p-Value
	Meng, X. 2013	1.580	0.871	2.867	1.506	0.132
	Dou, C. 2015	2.370	0.922	6.092	1.792	0.073
	Li, D. 2015	0.591	0.229	1.523	-1.089	0.276
	Tu, H. 2015	1.558	0.532	4.567	0.809	0.419
	Jiang, C. 2017	0.605	0.236	1.550	-1.046	0.295
	Tang, T.T. 2017	0.784	0.325	1.895	-0.539	0.590
	Wu, Z. 2017	0.755	0.232	2.457	-0.466	0.641
	Tong, Z. 2018	0.880	0.280	2.764	-0.219	0.827
	Wang, F. 2018	1.588	0.412	6.122	0.672	0.502
	Chen, J. 2019	0.542	0.205	1.432	-1.236	0.217
	Azar, M. 2020	3.333	0.505	22.017	1.250	0.211
	Kang, Y. 2020	0.415	0.079	2.195	-1.034	0.301
	Yuan, Z. 2020	0.661	0.221	1.979	-0.740	0.459
Fixed		1.012	0.766	1.337	0.086	0.931

Statistics for each study

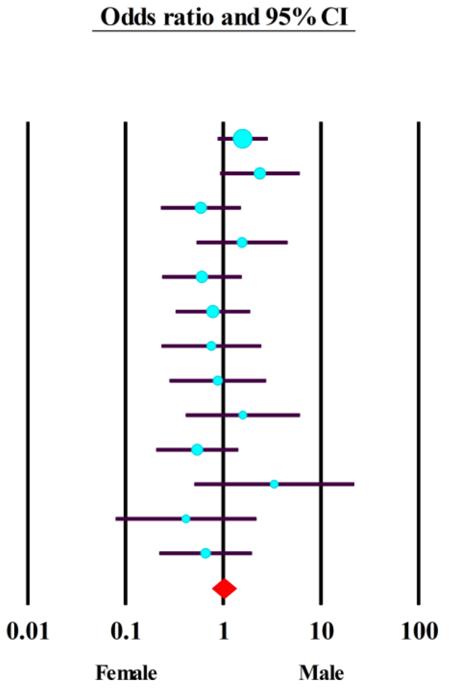

Figure S18- Forrest plot of low miR-212 expression in association with gender (male vs. female).

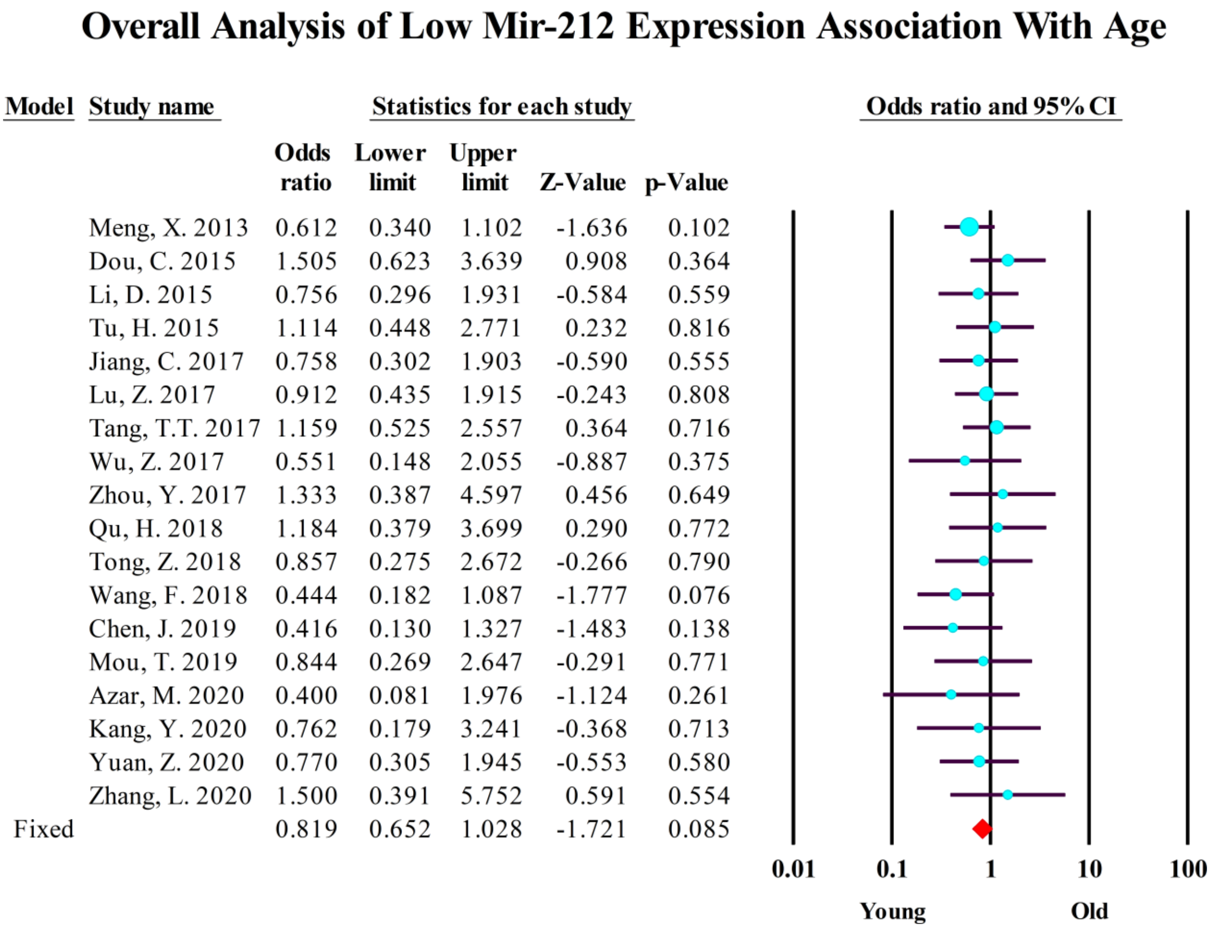

Model	Study name	Odds ratio	Lower limit	Upper limit	Z-Value	p-Value
	Meng, X. 2013	0.612	0.340	1.102	-1.636	0.102
	Dou, C. 2015	1.505	0.623	3.639	0.908	0.364
	Li, D. 2015	0.756	0.296	1.931	-0.584	0.559
	Tu, H. 2015	1.114	0.448	2.771	0.232	0.816
	Jiang, C. 2017	0.758	0.302	1.903	-0.590	0.555
	Lu, Z. 2017	0.912	0.435	1.915	-0.243	0.808
	Tang, T.T. 2017	1.159	0.525	2.557	0.364	0.716
	Wu, Z. 2017	0.551	0.148	2.055	-0.887	0.375
	Zhou, Y. 2017	1.333	0.387	4.597	0.456	0.649
	Qu, H. 2018	1.184	0.379	3.699	0.290	0.772
	Tong, Z. 2018	0.857	0.275	2.672	-0.266	0.790
	Wang, F. 2018	0.444	0.182	1.087	-1.777	0.076
	Chen, J. 2019	0.416	0.130	1.327	-1.483	0.138
	Mou, T. 2019	0.844	0.269	2.647	-0.291	0.771
	Azar, M. 2020	0.400	0.081	1.976	-1.124	0.261
	Kang, Y. 2020	0.762	0.179	3.241	-0.368	0.713
	Yuan, Z. 2020	0.770	0.305	1.945	-0.553	0.580
	Zhang, L. 2020	1.500	0.391	5.752	0.591	0.554
Fixed		0.819	0.652	1.028	-1.721	0.085

Figure S19- Forrest plot of low miR-212 expression in association with age (old vs. young).

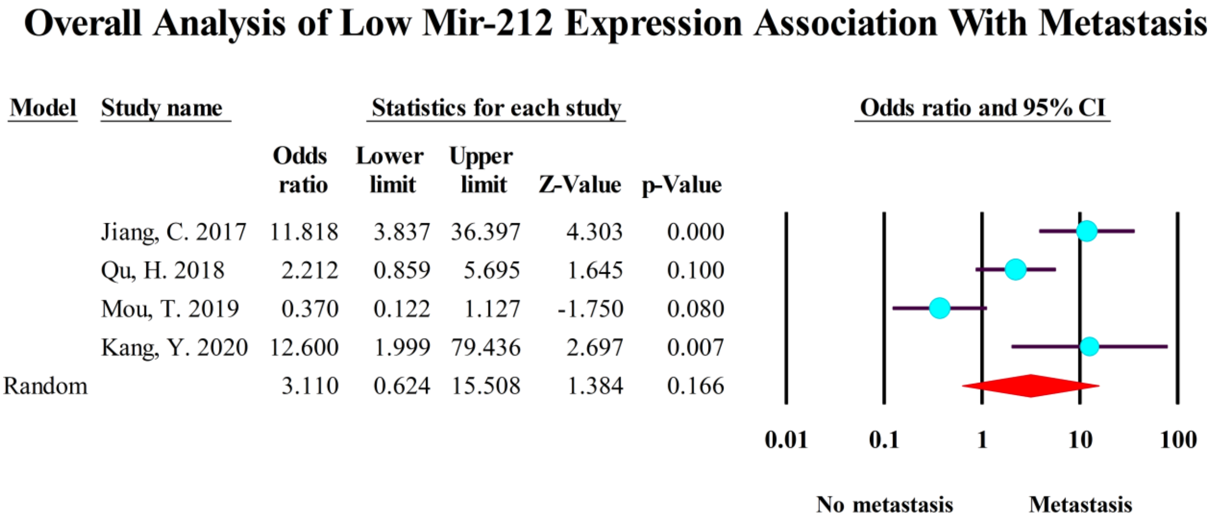

Model	Study name	Odds ratio	Lower limit	Upper limit	Z-Value	p-Value
	Jiang, C. 2017	11.818	3.837	36.397	4.303	0.000
	Qu, H. 2018	2.212	0.859	5.695	1.645	0.100
	Mou, T. 2019	0.370	0.122	1.127	-1.750	0.080
	Kang, Y. 2020	12.600	1.999	79.436	2.697	0.007
Random		3.110	0.624	15.508	1.384	0.166

Figure S20- Forrest plot of low miR-212 expression in association with metastasis (yes vs. no).

Overall Analysis of Low Mir-212 Expression Association With Lymphatic Metastasis

Model	Study name	Odds ratio	Lower limit	Upper limit	Z-Value	p-Value
	Tang, T.T. 2017	4.663	1.814	11.983	3.197	0.001
	Tong, Z. 2018	49.000	2.697	890.351	2.630	0.009
	Azar, M. 2020	0.400	0.022	7.201	-0.621	0.534
Random		4.529	0.576	35.588	1.436	0.151

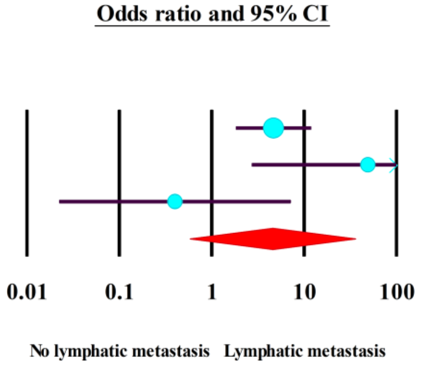

Figure S21- Forrest plot of low miR-212 expression in association with lymphatic metastasis (yes vs. no).

Overall Analysis of Low Mir-212 Expression Association With Tumor Size

Model	Study name	Odds ratio	Lower limit	Upper limit	Z-Value	p-Value
	Meng, X. 2013	0.816	0.436	1.525	-0.638	0.524
	Dou, C. 2015	2.769	1.174	6.531	2.327	0.020
	Li, D. 2015	1.950	0.725	5.248	1.322	0.186
	Tu, H. 2015	3.606	1.398	9.299	2.654	0.008
	Lu, Z. 2017	2.427	1.151	5.118	2.329	0.020
	Wu, Z. 2017	0.278	0.076	1.015	-1.937	0.053
	Qu, H. 2018	4.545	1.686	12.251	2.993	0.003
	Tong, Z. 2018	7.389	1.748	31.225	2.720	0.007
	Wang, F. 2018	0.310	0.122	0.789	-2.459	0.014
	Chen, J. 2019	0.530	0.215	1.304	-1.383	0.167
	Mou, T. 2019	0.364	0.119	1.109	-1.777	0.076
	Yue, H. 2019	0.074	0.012	0.461	-2.790	0.005
	Azar, M. 2020	0.351	0.070	1.761	-1.273	0.203
	Yuan, Z. 2020	7.273	2.834	18.667	4.126	0.000
Random		1.195	0.635	2.249	0.553	0.580

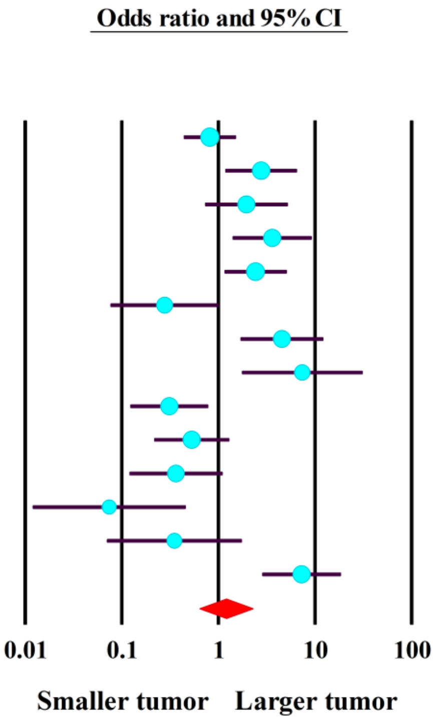

Figure S22- Forrest plot of low miR-212 expression in association with tumor size (large vs. small).

Overall Analysis of Low Mir-212 Expression Association With Tumor Size (Cut-Off=5cm)

Model	Study name	Odds ratio	Lower limit	Upper limit	Z-Value	p-Value
	Meng, X. 2013	0.816	0.436	1.525	-0.638	0.524
	Dou, C. 2015	2.769	1.174	6.531	2.327	0.020
	Tu, H. 2015	3.606	1.398	9.299	2.654	0.008
	Tong, Z. 2018	7.389	1.748	31.225	2.720	0.007
	Wang, F. 2018	0.310	0.122	0.789	-2.459	0.014
	Chen, J. 2019	0.530	0.215	1.304	-1.383	0.167
	Mou, T. 2019	0.364	0.119	1.109	-1.777	0.076
	Yuan, Z. 2020	7.273	2.834	18.667	4.126	0.000
Random		1.455	0.630	3.364	0.878	0.380

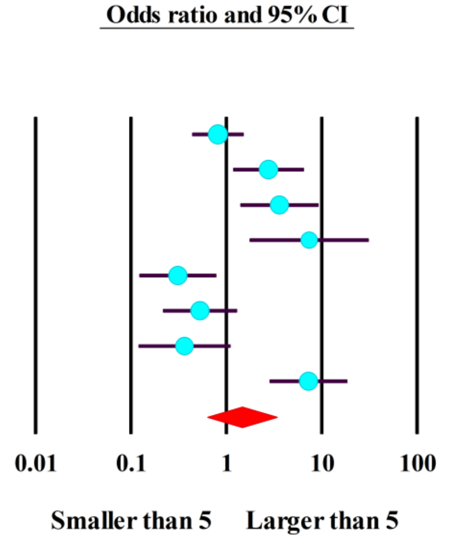

Figure S23- Forrest plot of low miR-212 expression in association with tumor size ([>5 or ≥5] vs. [<5 or ≤5]).

Overall Analysis of Low Mir-212 Expression Association With T Stage

Model	Study name	Odds ratio	Lower limit	Upper limit	Z-Value	p-Value
	Meng, X. 2013	1.181	0.549	2.543	0.426	0.670
	Wu, Z. 2017	0.180	0.041	0.787	-2.278	0.023
Random		0.515	0.082	3.212	-0.711	0.477

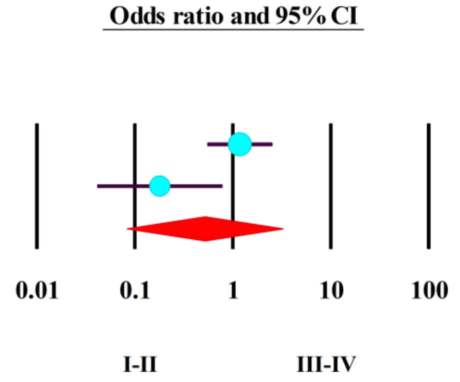

Figure S24- Forrest plot of low miR-212 expression in association with T stage (III-IV vs. I-II).

Overall Analysis of Low MiR-212 Expression Association With TNM Stage

Model	Study name	Odds ratio	Lower limit	Upper limit	Z-Value	p-Value
	Dou, C. 2015	2.593	1.074	6.256	2.120	0.034
	Tu, H. 2015	3.703	1.347	10.179	2.537	0.011
	Jiang, C. 2017	3.925	1.377	11.186	2.559	0.011
	Tang, T.T. 2017	3.494	1.490	8.195	2.876	0.004
	Wu, Z. 2017	0.409	0.118	1.421	-1.407	0.159
	Tong, Z. 2018	21.000	2.450	179.984	2.778	0.005
	Wang, F. 2018	3.889	1.533	9.868	2.859	0.004
	Mou, T. 2019	3.284	1.059	10.186	2.059	0.040
	Yue, H. 2019	0.283	0.052	1.533	-1.464	0.143
	Azar, M. 2020	1.714	0.278	10.589	0.580	0.562
	Kang, Y. 2020	6.000	1.125	31.989	2.098	0.036
	Yuan, Z. 2020	3.750	1.486	9.465	2.798	0.005
Random		2.719	1.674	4.415	4.044	0.000

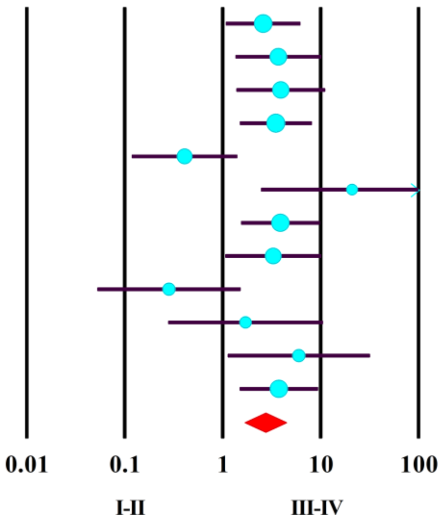

Figure S25- Forrest plot of low miR-212 expression in association with TNM stage (III-IV vs. I-II).

Overall Analysis of Low MiR-212 Expression Association With Differentiation

Model	Study name	Odds ratio	Lower limit	Upper limit	Z-Value	p-Value
	Li, D. 2015	0.845	0.333	2.145	-0.354	0.723
	Lu, Z. 2017	1.035	0.505	2.118	0.094	0.925
	Wang, F. 2018	0.588	0.237	1.460	-1.144	0.253
	Azar, M. 2020	5.714	0.446	73.191	1.340	0.180
Fixed		0.894	0.557	1.435	-0.465	0.642

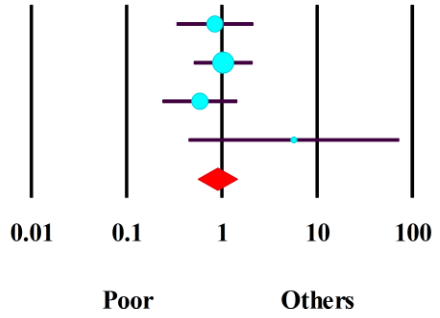

Figure S26- Forrest plot of low miR-212 expression in association with differentiation (others vs. poor).

Overall Analysis of Low MiR-212 Expression Association With Hepatitis B Virus

Model	Study name	Odds ratio	Lower limit	Upper limit	Z-Value	p-Value
		**Statistics for each study**				
	Dou, C. 2015	0.557	0.233	1.335	-1.312	0.190
	Tu, H. 2015	0.663	0.271	1.619	-0.903	0.367
	Wang, F. 2018	1.926	0.695	5.335	1.261	0.207
	Chen, J. 2019	2.929	0.535	16.042	1.238	0.216
	Yuan, Z. 2020	0.848	0.274	2.624	-0.285	0.775
Random		0.955	0.545	1.673	-0.161	0.872

Odds ratio and 95% CI

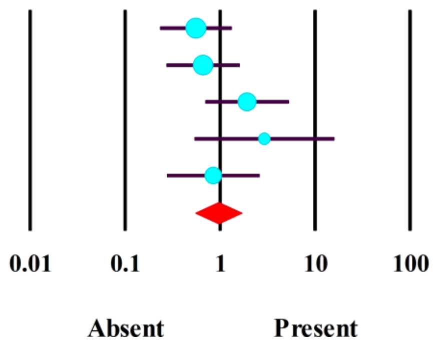

Figure S27- Forrest plot of low miR-212 expression in association with hepatitis B virus (HBV) (present vs. absent).

Overall Analysis of Low miR-212 Expression Association With Serum AFP Level (High/Low)

Model	Study name	Odds ratio	Lower limit	Upper limit	Z-Value	p-Value
		**Statistics for each study**				
	Dou, C. 2015	2.500	1.071	5.834	2.119	0.034
	Tu, H. 2015	3.345	1.346	8.312	2.601	0.009
	Wang, F. 2018	1.385	0.554	3.458	0.697	0.486
	Chen, J. 2019	1.869	0.781	4.474	1.405	0.160
	Yuan, Z. 2020	3.088	1.253	7.606	2.451	0.014
Fixed		2.319	1.559	3.451	4.150	0.000

Odds ratio and 95% CI

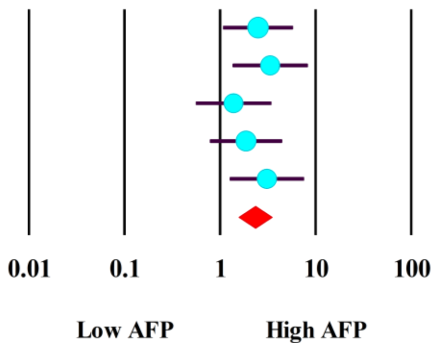

Figure S28- Forrest plot of low miR-212 expression in association with serum AFP level (high vs. low).

Overall Analysis of Low MiR-212 Expression Association With Serum AFP Level (Cut-Off=200)

Model	Study name	Odds ratio	Lower limit	Upper limit	Z-Value	p-Value
	Wang, F. 2018	1.385	0.554	3.458	0.697	0.486
	Chen, J. 2019	1.869	0.781	4.474	1.405	0.160
Fixed		1.620	0.862	3.047	1.498	0.134

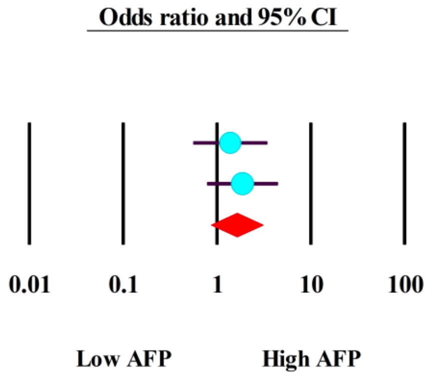

Figure S29- Forrest plot of low miR-212 expression in association with serum AFP level (>200 vs. ≤200).

Overall Analysis of Low MiR-212 Expression Association With Serum AFP Level (Cut-Off=400)

Model	Study name	Odds ratio	Lower limit	Upper limit	Z-Value	p-Value
	Dou, C. 2015	2.500	1.071	5.834	2.119	0.034
	Tu, H. 2015	3.345	1.346	8.312	2.601	0.009
	Yuan, Z. 2020	3.088	1.253	7.606	2.451	0.014
Fixed		2.933	1.759	4.889	4.127	0.000

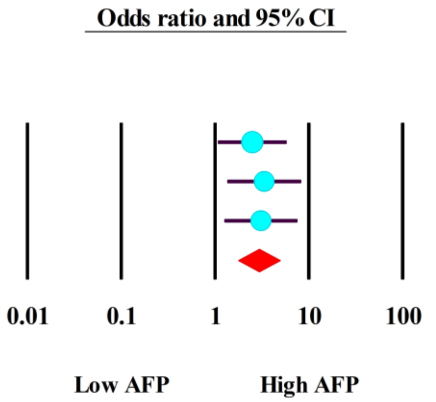

Figure S30- Forrest plot of low miR-212 expression in association with serum AFP level (≥400 vs. <400).

Overall Analysis of Low MiR-212 Expression Association With Number of Tumor Nodules

Model	Study name	Odds ratio	Lower limit	Upper limit	Z-Value	p-Value
	Dou, C. 2015	0.577	0.251	1.327	-1.294	0.196
	Tu, H. 2015	1.694	0.613	4.682	1.015	0.310
	Chen, J. 2019	2.292	0.916	5.735	1.773	0.076
Random		1.275	0.536	3.031	0.550	0.583

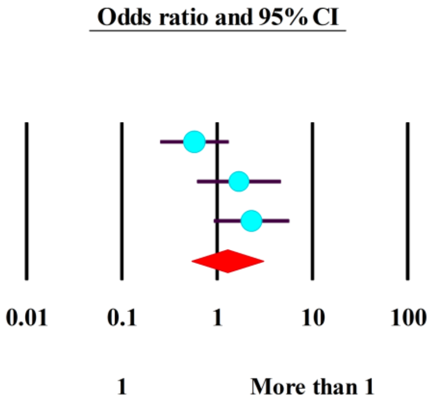

Figure S31- Forrest plot of low miR-212 expression in association with number of tumor nodules (≥2 vs. 1).

Overall Analysis of Low MiR-212 Expression Association With Cirrhosis

Model	Study name	Odds ratio	Lower limit	Upper limit	Z-Value	p-Value
	Dou, C. 2015	0.789	0.349	1.786	-0.569	0.570
	Tu, H. 2015	0.752	0.319	1.770	-0.653	0.514
	Wang, F. 2018	2.385	0.940	6.052	1.830	0.067
	Chen, J. 2019	1.589	0.603	4.186	0.937	0.349
	Yuan, Z. 2020	0.943	0.397	2.243	-0.132	0.895
Fixed		1.111	0.748	1.649	0.522	0.602

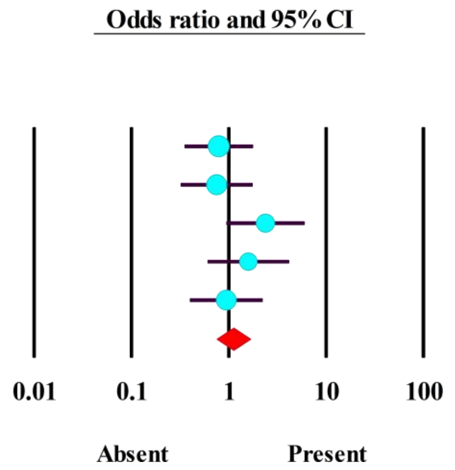

Figure S32- Forrest plot of low miR-212 expression in association with cirrhosis (present vs. absent).

Overall Analysis of Low MiR-212 Expression Association With Venous Infltration

Model	Study name	Odds ratio	Lower limit	Upper limit	Z-Value	p-Value
	Dou, C. 2015	1.885	0.834	4.259	1.524	0.127
	Tu, H. 2015	1.453	0.621	3.396	0.862	0.389
	Yuan, Z. 2020	2.354	0.999	5.548	1.957	0.050
Fixed		1.859	1.145	3.019	2.506	0.012

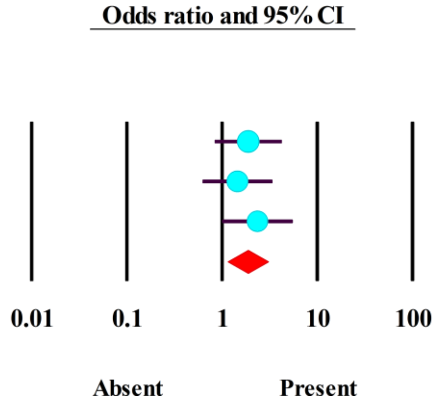

Figure S33- Forrest plot of low miR-212 expression in association with venous infiltration (present vs. absent).

Overall Analysis of Low MiR-212 Expression Association With Edmondson-Steiner Grade

Model	Study name	Odds ratio	Lower limit	Upper limit	Z-Value	p-Value
	Dou, C. 2015	1.607	0.682	3.787	1.085	0.278
	Tu, H. 2015	2.915	1.201	7.073	2.366	0.018
	Chen, J. 2019	1.158	0.489	2.741	0.333	0.739
	Yuan, Z. 2020	1.818	0.708	4.667	1.243	0.214
Fixed		1.757	1.129	2.735	2.496	0.013

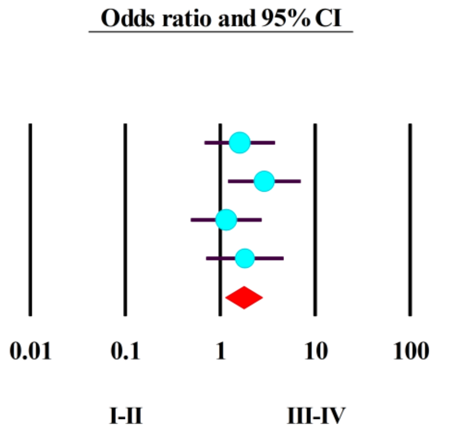

Figure S34- Forrest plot of low miR-212 expression in association with Edmondson-Steiner grade (III-IV vs. I-II).

Overall Analysis of Low MiR-212 Expression Association With Venous Invasion

Model	Study name	Odds ratio	Lower limit	Upper limit	Z-Value	p-Value
	Li, D. 2015	1.838	0.668	5.056	1.180	0.238
	Lu, Z. 2017	3.301	1.568	6.950	3.144	0.002
	Chen, J. 2019	4.356	1.732	10.954	3.127	0.002
	Yue, H. 2019	0.208	0.047	0.921	-2.069	0.039
Random		1.772	0.629	4.996	1.082	0.279

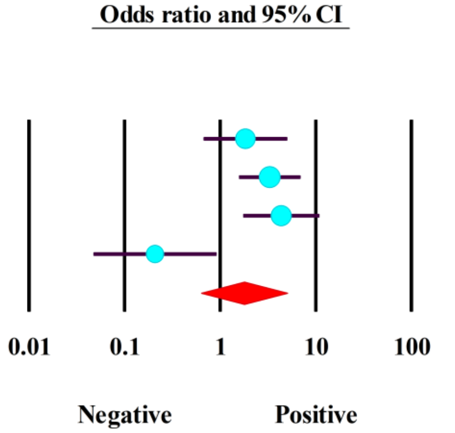

Figure S35- Forrest plot of low miR-212 expression in association with venous invasion (positive vs. negative).

Overall Analysis of Low MiR-212 Expression Association With Distant Metastasis

Model	Study name	Odds ratio	Lower limit	Upper limit	Z-Value	p-Value
	Meng, X. 2013	3.273	1.424	7.522	2.792	0.005
	Wu, Z. 2017	0.800	0.184	3.473	-0.298	0.766
	Zhou, Y. 2017	0.110	0.022	0.559	-2.662	0.008
Random		0.726	0.102	5.183	-0.319	0.750

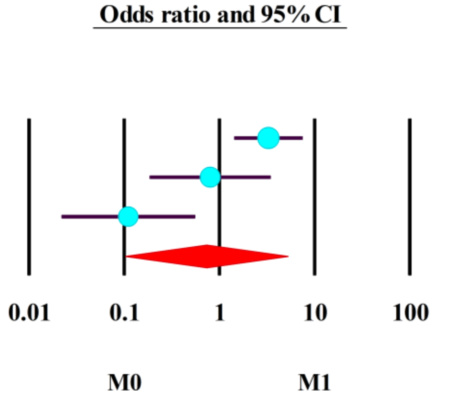

Figure S36- Forrest plot of low miR-212 expression in association with distant metastasis (M1 vs. M0).

Overall Analysis of Low MiR-212 Expression Association With Histological Grade

Model	Study name	Odds ratio	Lower limit	Upper limit	Z-Value	p-Value
		**Statistics for each study**				
	Meng, X. 2013	1.538	0.770	3.075	1.219	0.223
	Wu, Z. 2017	0.551	0.148	2.055	-0.887	0.375
Fixed		1.231	0.667	2.273	0.666	0.505

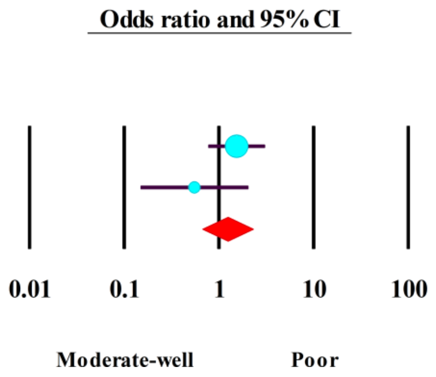

Figure S37- Forrest plot of low miR-212 expression in association with histological grade (poor vs. moderate-well).

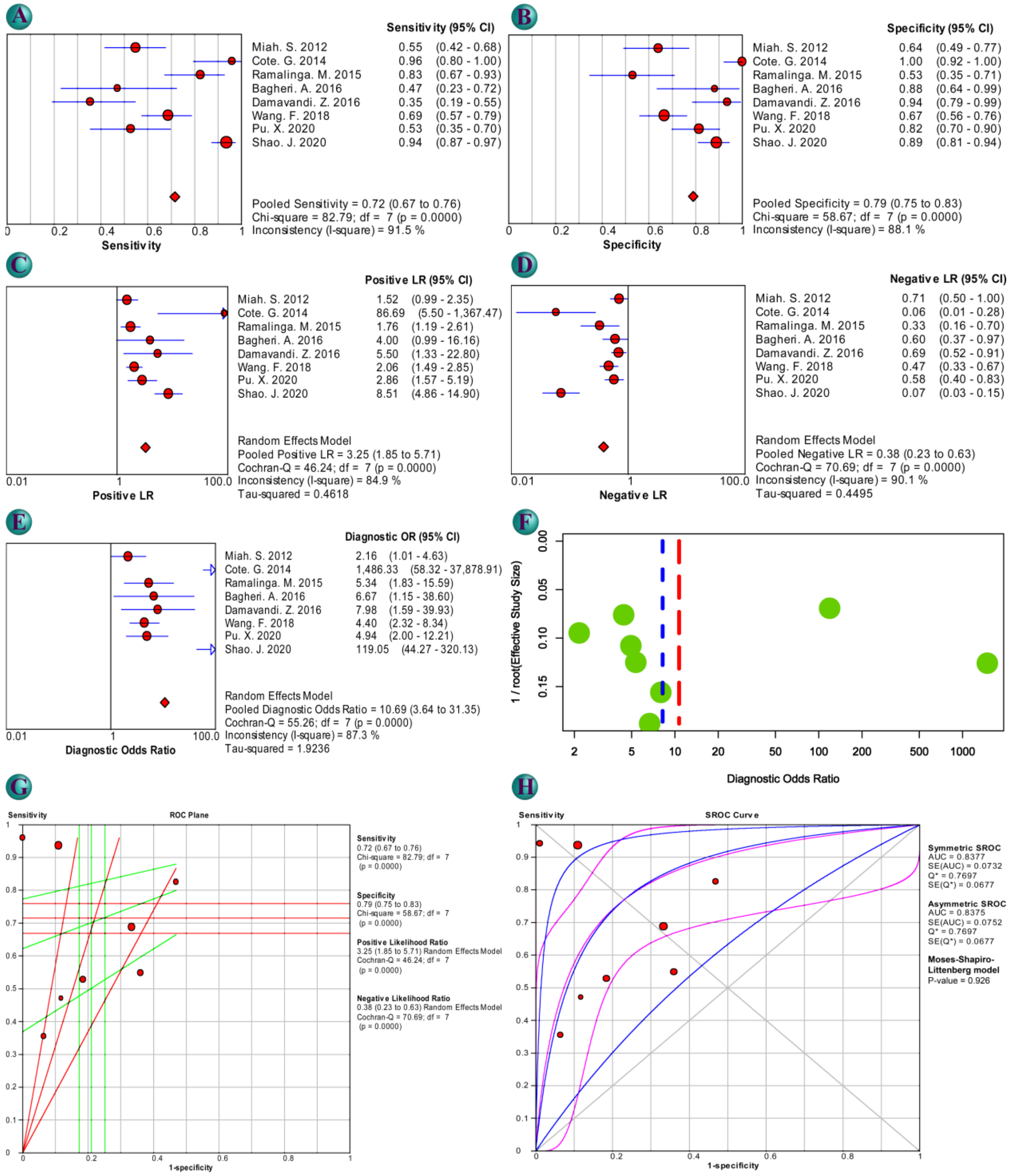

Figure S38- Diagnostic evaluation of cancer using miR-212 level as determinant based on numbers preferably obtained by Youden Index: Forrest plot of sensitivity (A), specificity (B), positive likelihood ratio (LR) (C), negative LR (D), and diagnostic odds ratio (OR) (E), Deeks' funnel plot (F), receiver operating characteristic (ROC) plane (G) and Summary ROC (SROC) curve (H).

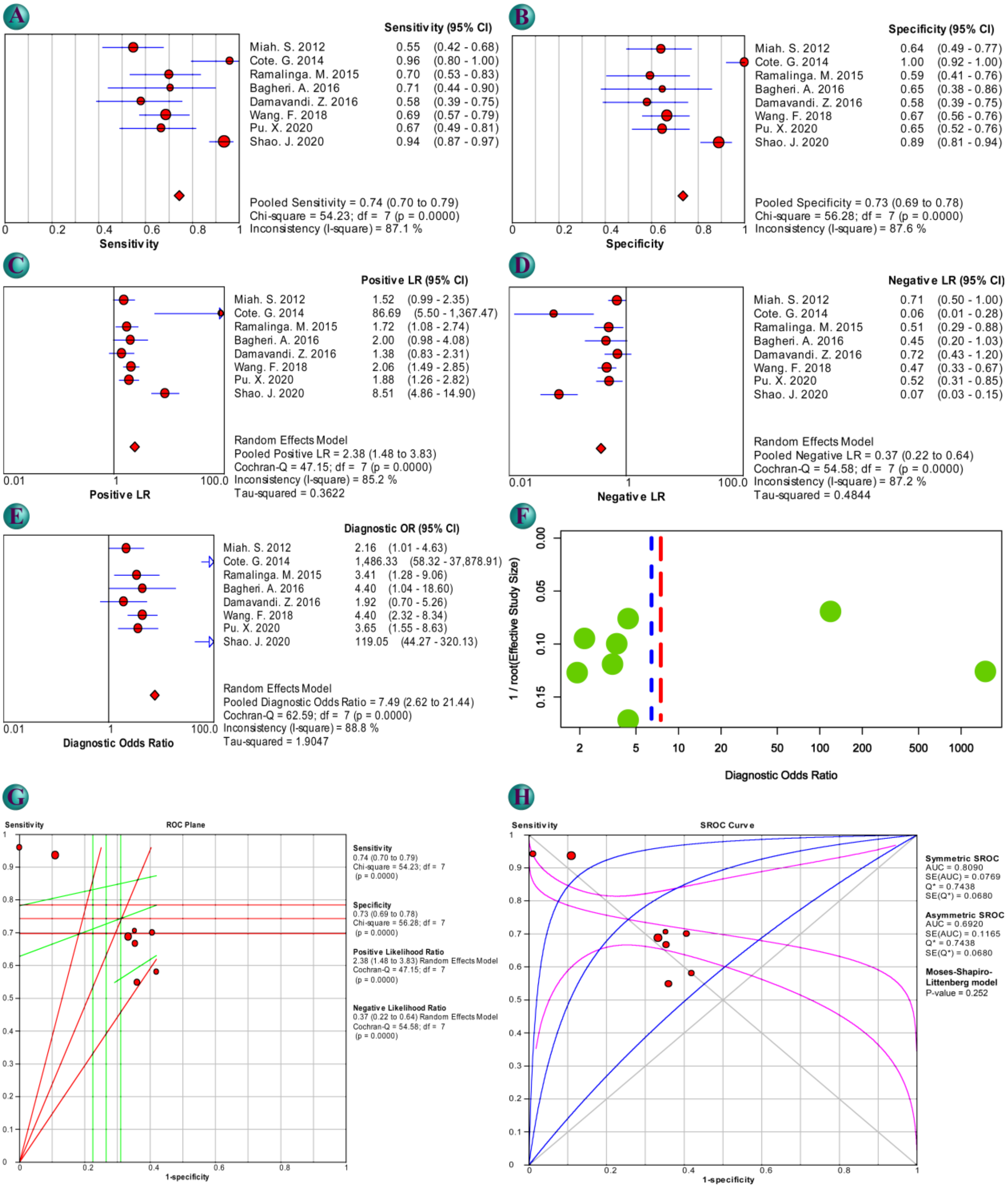

Figure S39- Diagnostic evaluation of cancer using miR-212 level as determinant based on numbers preferably obtained by Index of Union: Forrest plot of sensitivity (A), specificity (B), positive likelihood ratio (LR) (C), negative LR (D), and diagnostic odds ratio (OR) (E), Deeks' funnel plot (F), receiver operating characteristic (ROC) plane (G) and Summary ROC (SROC) curve (H).

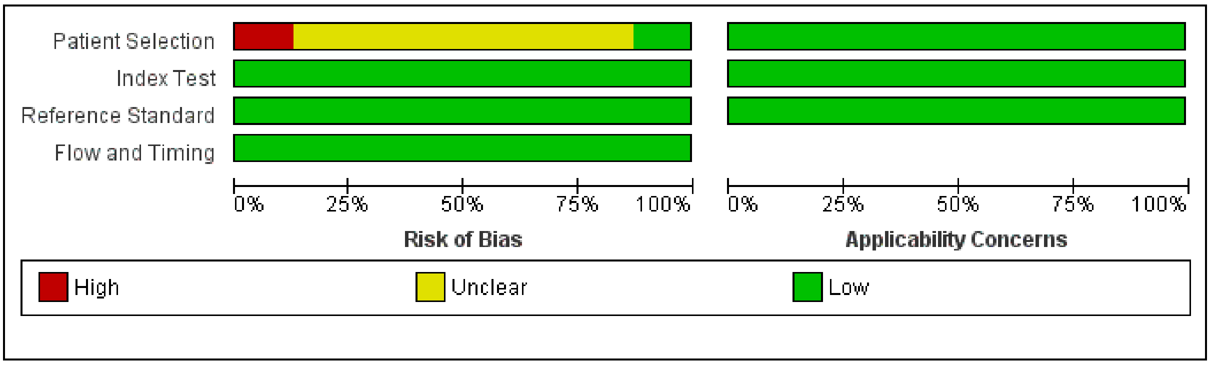

Figure S40- Methodological quality graph of studies included in the diagnostic evaluation.

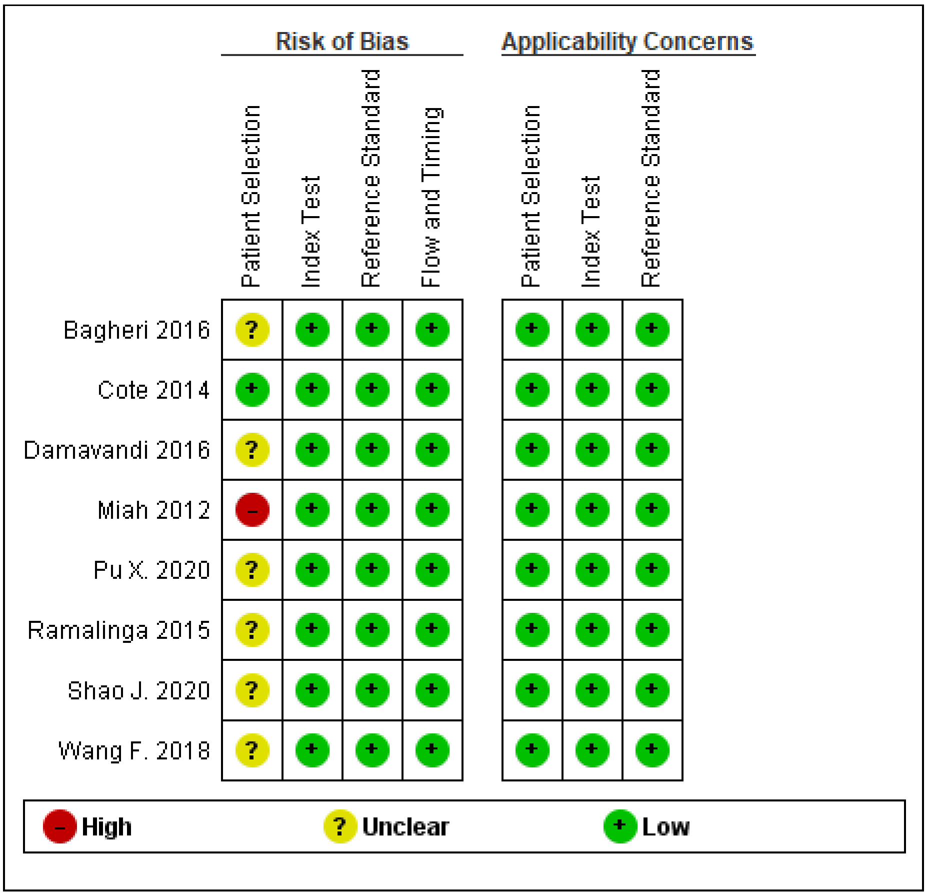

Figure S41- Methodological quality summary of studies included in the diagnostic evaluation.

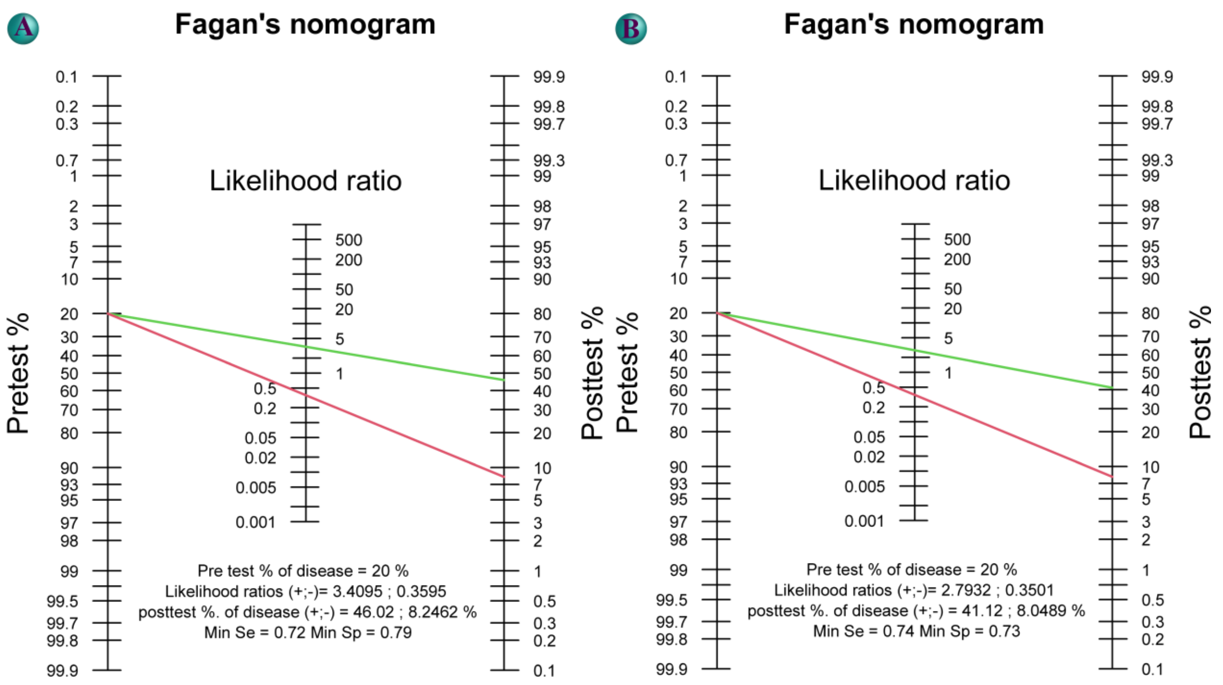

Figure S42- Fagan's nomogram based on pooled sensitivity and specificity using numbers preferably obtained by Youden Index (A) and Index of Union (B).

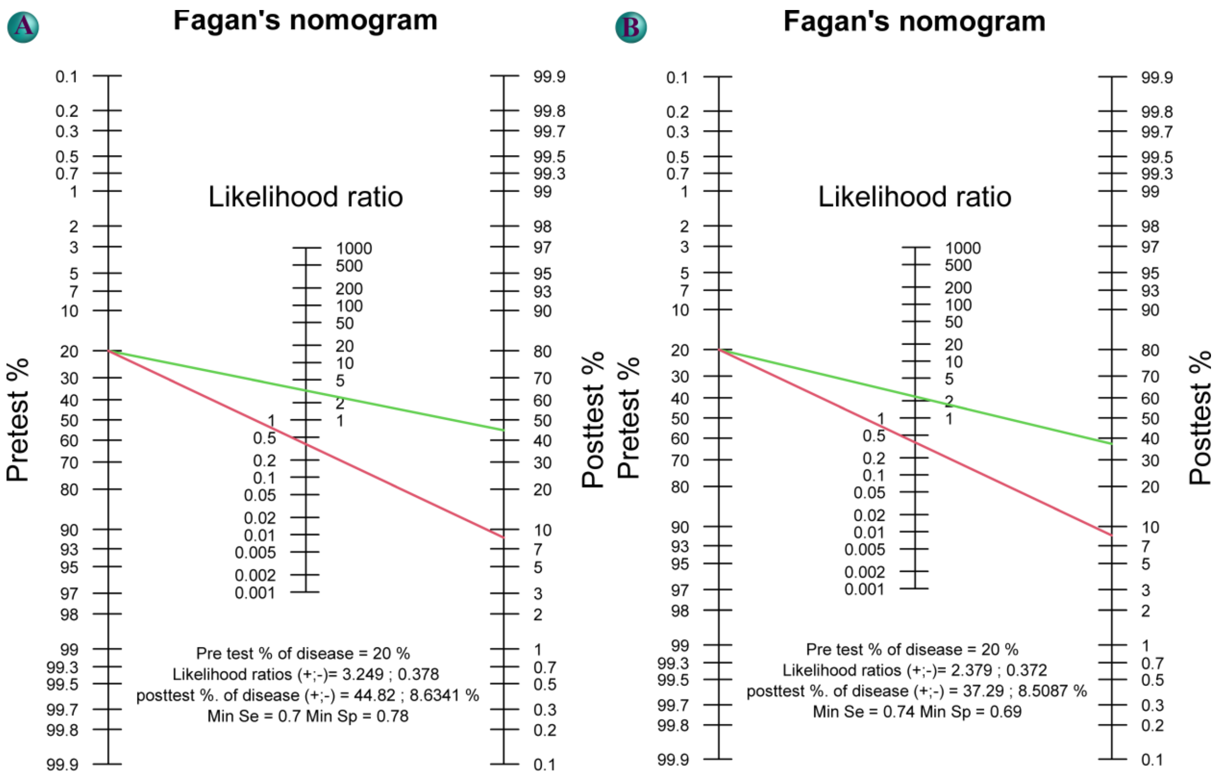

Figure S43- Fagan's nomogram based on pooled positive and negative likelihood ratios using numbers preferably obtained by Youden Index (A) and Index of Union (B).
